# Differential Sensitivity of Mindfulness Questionnaires to Change With Treatment: A Systematic Review and Meta-Analysis

**DOI:** 10.1037/pas0000744

**Published:** 2019-08-01

**Authors:** Ruth Baer, Jenny Gu, Kate Cavanagh, Clara Strauss

**Affiliations:** 1 Department of Psychology, University of Kentucky, and Department of Psychiatry, University of Oxford; 2 School of Psychology, University of Sussex; 3 School of Psychology, University of Sussex, and Sussex Partnership NHS Foundation Trust, Swandean, United Kingdom

**Keywords:** mindfulness, self-report assessment, differential sensitivity to change with treatment, meta-analysis, randomized trials

## Abstract

In support of the construct validity of mindfulness questionnaires, meta-analytic reviews have reported that scores increase in mindfulness-based interventions (MBIs). However, several studies have also found increased mindfulness scores in interventions with no explicit mindfulness training, raising a question about differential sensitivity to change with treatment. We conducted a systematic review and meta-analysis of 37 randomized controlled trials in which mindfulness questionnaires were administered before and after an evidence-based MBI and a nonmindfulness-based active control condition. The central question was whether increases in mindfulness scores would be greater in the MBI than in the comparison group. On average, participants in MBIs showed significantly greater pre–post changes in mindfulness scores than were seen in active control conditions with no explicit mindfulness elements, with a small overall effect size. This effect was moderated by which mindfulness questionnaire was used, by the type of active control condition, and by whether the MBI and control were matched for amount of session time. When mindfulness facet scores were analyzed separately, MBIs showed significantly greater pre–post increases than active controls in observing, nonjudging, and nonreactivity but not in describing or acting with awareness. Although findings provide partial support for the differential sensitivity of mindfulness questionnaires to change with treatment, the nonsignificant difference in pre–post change when the MBI and control were matched for session time highlights the need to clarify how mindfulness skills are acquired in MBIs and in other interventions and whether revisions to mindfulness questionnaires would increase their specificity to changes in mindfulness skills.

## Public Significance Statement

This review found that scores on mindfulness questionnaires increase more in mindfulness-based interventions than in interventions with no explicit mindfulness training, but not under all conditions. When the two treatments had equal session time, increases in mindfulness scores were similar. Nonmindfulness-based interventions may implicitly cultivate mindfulness skills, or the questionnaires may need revisions to increase their specificity to changes in mindfulness skills.

In the psychological literature, *mindfulness* is typically defined as a particular form of present-focused attention or awareness. Many descriptions include two general elements: the attention itself and the qualities of the attention. Examples of these two elements, sometimes called the *what* and the *how* of mindfulness (Linehan, 1993), are shown in Table 1 and suggest that mindfulness involves paying attention to the present moment with qualities of openness, nonjudgment, acceptance, friendliness, curiosity, kindness, and compassion.[Table-anchor tbl1]

The assessment of mindfulness is important in understanding its relationships with other variables and its role in health and wellbeing (Baer, 2011; Park, Reilly-Spong, & Gross, 2013; Quaglia, Braun, Freeman, McDaniel, & Brown, 2016). Measurement of mindfulness relies largely on self-report questionnaires designed to assess a general disposition or trait-like tendency to be mindful in daily life. This tendency is understood to vary in the general population and to be susceptible to change with training and practice. The most commonly used mindfulness questionnaires are the Mindful Attention Awareness Scale (MAAS: Brown & Ryan, 2003), the Five Facet Mindfulness Questionnaire (FFMQ; Baer, Smith, Hopkins, Krietemeyer, & Toney, 2006), the Kentucky Inventory of Mindfulness Skills (KIMS; Baer, Smith, & Allen, 2004), the Freiburg Mindfulness Inventory (FMI; Buchheld, Grossman, & Walach, 2001), and the Cognitive Affective Mindfulness Scale—Revised (CAMS–R; Hayes & Feldman, 2004).

The psychometric properties of these questionnaires have been widely studied. In a review, Park et al. (2013) described evidence for their internal consistency as strong, meaning that multiple studies of good quality have reported Cronbach’s alphas ≥.70 for unidimensional scales or subscales. Test–retest reliability has been examined less often. Park et al. reported adequate values for three of four KIMS subscales (intraclass correlation [ICC] ≥ .70 or Pearson’s *r* ≥ .80). Mixed findings have been reported for the FMI, with an unspecified coefficient of .67 in a Chinese sample (Chen & Zhou, 2014) and ICC = .80 in a French sample (Trousselard et al., 2010). The most comprehensive studies of test–retest reliability were reported by Jensen, Krogh, Westphael, and Hjordt (2019) and Jensen, Niclasen, Vangkilde, Petersen, and Hasselbalch (2016), who examined both the MAAS and the FFMQ in Danish student and community samples. Test-retest reliabilities were good over a 2-week interval (MAAS: ICC = .88, FFMQ: ICCs ≥ .82 for all facets), and satisfactory over a 6-month interval (MAAS: ICC = .74, FFMQ: ICCs ≥ .74 for all facets). Both instruments showed greater 6-month stability than was seen for a measure of psychological distress.

Clear unidimensional factor structures have been demonstrated for the MAAS (Jensen et al., 2016) and CAMS–R (Hayes & Feldman, 2004). The KIMS and FFMQ have multidimensional factor structures that differ for meditators and nonmeditators but are generally consistent within these groups (Baer et al., 2008; Christopher, Neuser, Michael, & Baitmangalkar, 2012; Curtiss & Klemanski, 2014; Gu et al., 2016; Williams, Dalgleish, Karl, & Kuyken, 2014). Park et al. (2013) reported that construct validity through hypothesis testing (e.g., whether mindfulness scores correlate in predicted ways with other measures and differ as expected between groups) was strong for the MAAS, KIMS, CAMS–R, and FFMQ and mixed for the FMI. Meta-analyses have shown that scores on mindfulness questionnaires increase in response to mindfulness training (Khoury et al., 2013; Quaglia et al., 2016; Visted, Vollestad, Nielsen, & Nielsen, 2015), and that therapeutic effects of the two most studied MBIs (mindfulness-based stress reduction [MBSR; Kabat-Zinn, 1982] and mindfulness-based cognitive therapy [MBCT; Segal, Williams, & Teasdale, 2013]) appear to be mediated by increases in self-reported mindfulness skills (Gu, Strauss, Bond, & Cavanagh, 2015).

Overall, mindfulness questionnaires have performed reasonably well on a variety of psychometric tests. However, a question has arisen about their differential sensitivity to change with intervention, with several studies showing that mindfulness scores increased about equally in MBIs and in other active treatments. For example, Goldberg et al. (2016) found that FFMQ scores showed similar increases in MBSR and in the Health Enhancement Program (HEP; MacCoon et al., 2012), an active control designed to match many aspects of MBSR (group size, session time, home practice, etc.) while including no mindfulness training. Both groups showed larger increases in FFMQ scores than were seen in a waitlist control group. In a meta-analysis, Visted et al. (2015) found no significant differences in mindfulness scores between mindfulness training and active control groups. In contrast, other studies have shown higher posttreatment mindfulness scores in MBIs than in other treatments. In adults with generalized anxiety disorder, Hoge et al. (2015) reported higher mindfulness scores in MBSR than in a stress management education group. Johns et al. (2016) found similar results in adults with cancer and fatigue.

To investigate these conflicting findings, we conducted a systematic review and meta-analysis of differential sensitivity of mindfulness questionnaires to change with intervention. We included only randomized controlled trials that compared an evidence-based MBI to an active control with no explicit mindfulness training. We hypothesized that mindfulness questionnaires would show greater pre–post increases in MBIs than in active controls. Confirmation of this hypothesis would add to the evidence supporting the construct validity of mindfulness questionnaires by showing that scores increase as expected with mindfulness training but not with other types of intervention. Disconfirmation of the hypothesis (i.e., similar changes in mindfulness scores for MBIs and comparison conditions) might suggest that the questionnaires, though written to be specific to mindfulness skills, are sensitive to changes in other constructs, such as distress, that improve with a variety of interventions. Alternatively, other programs may implicitly teach mindfulness or related skills such as awareness of thoughts and feelings and willingness to experience them.

Differential sensitivity to change with treatment can be tested more clearly when there is a high level of confidence that the MBI should teach mindfulness skills. For this reason, we included only studies of MBSR, MBCT, and well-established variants that have a strong evidence based and are consistent with the defining features of MBIs as described by Crane et al. (2017). These features include intensive training in mindfulness meditation through in-session and home practice over several weeks, an experiential inquiry-based learning process, and other exercises designed to help participants develop a new relationship to present-moment experience based on friendly interest, decentering, equanimity, and compassion (see Crane et al. for more detail). Exclusion of single-session and laboratory-based mindfulness inductions and other mindfulness trainings with little empirical support provides a clearer test of the hypothesis by strengthening the expectation that the MBI should lead to increased mindfulness skills and minimizing the possibility that the two interventions yielded similar mindfulness scores because of inadequate mindfulness teaching in the MBI.

We expected that differential sensitivity of mindfulness questionnaires to change with treatment could be influenced by aspects of the questionnaires themselves, aspects of the comparison treatments, or the design of the trials in which the questionnaires were used. We conducted planned moderator analyses for four such variables. First, measures differ in their conceptualization of mindfulness and some may have better differential sensitivity to change with intervention than others; therefore, we examined whether findings differed depending on which mindfulness measure was used. Second, we expected that the type of active control intervention could affect the extent to which mindfulness questionnaires show differing levels of change in the two groups. Some comparison treatments might cultivate mindfulness-related skills, such as awareness of thoughts and feelings and willingness to experience them, even if they include no explicit mindfulness training. In particular, cognitive–behavioral therapy is known to cultivate decentering, which is strongly correlated with mindfulness (Carmody, Baer, Lykins, & Olendzki, 2009) and improves in both CBT and MBIs (Carmody et al., 2009; Farb et al., 2018; Fresco, Segal, Buis, & Kennedy, 2007). We predicted that differences between interventions in cultivation of mindfulness skills would be smaller when comparing MBIs to CBT but larger when comparing MBIs to medication, which is not intended to teach skills, and larger when comparing MBIs to psychosocial interventions that are not designed to teach mindfulness or decentering.

Another aspect of the control intervention that might influence differential sensitivity to change with treatment is whether it is matched to the MBI for number and duration of sessions. If mindfulness questionnaires show differential sensitivity to change when session time is matched, it would suggest that the questionnaires measure something that changes with mindfulness training but not with treatments that provide equal time for the development of other skills or nonspecific factors such as support. On the other hand, if differential sensitivity is seen only when the MBI has greater session time, the possibility would remain that the non-MBI might have led to similar increases in mindfulness scores if more session time had been provided. This would suggest either that the questionnaires are sensitive to change in constructs other than mindfulness skills, or that both treatments cultivate mindfulness skills. Therefore, we examined whether matching for session time moderated the findings. Finally, cultivation of mindfulness skills may be stronger when the mindfulness training adheres to an evidence-based protocol. All included studies used MBIs with well-established protocols, but not all included fidelity checks; therefore, we examined whether findings differed depending on whether the study included a formal check of fidelity to the MBI protocol (with fidelity checks used as a proxy for quality of protocol adherence). A significant moderation would support the differential sensitivity of the mindfulness questionnaires by showing that scores increase more when there was an indication that mindfulness skills were well taught.

Our review focused only on differential sensitivity to change with treatment and did not address other aspects of validity which have been reviewed elsewhere. We did not analyze effects on clinical outcomes because we assumed that MBIs should teach mindfulness skills regardless of whether the intervention led to clinically meaningful reductions in symptoms, and because numerous meta-analyses examining the effects of MBIs on clinical outcomes are available. We included only measures of mindfulness and did not include measures of decentering, self-compassion, or other related constructs, which have been used less often in trials of MBIs.

Our review adds to previous meta-analyses in several ways. Khoury et al. (2013) focused primarily on clinical outcomes and did not examine differences between mindfulness questionnaires in sensitivity to change. Quaglia et al. (2016) collapsed across questionnaires to test common dimensions of mindfulness rather than examining each questionnaire separately and did not consider differences between types of active controls. Visted et al. (2015) did not exclude active controls with explicit mindfulness elements and included only 12 studies, whereas we found 37 comparing an MBI to an active control. Our review is unique in including only MBIs based on the gold standard curriculums of MBSR, MBCT or close variants, which are intensive courses designed to teach mindfulness skills. Our review is also unique in testing differences between mindfulness questionnaires and the effects of different types of active control groups on differential sensitivity to change with treatment.

## Method

The protocol for this meta-analysis was registered on PROSPERO (Registration Number: CRD42017065786) and conducted in accordance with the PRISMA guidelines (Moher, Liberati, Tetzlaff, & Altman, 2009).

### Search Strategy

The following databases were searched for studies up to 12 December 2017: PsycINFO, Scopus, Web of Science, and Medline. Abstracts or titles were searched using the following search term: (“mindfulness-based” OR MBCT OR MBSR OR Breathworks OR MBLC OR MBCP OR MBRP) AND random*. Clinical trial registers (ClinicalTrials.gov, ISRCTN.com) were also searched, using the search term *mindfulness*, to identify unpublished, completed interventional studies of MBIs which recruited adults. Corresponding authors of the final set of papers were e-mailed for any additional unpublished data (e.g., facet scores in addition to total scores), if sufficient data were not reported (e.g., papers reporting only baseline data), and for clarification (e.g., on number of participants in each condition). When authors failed to respond to the initial request for data, a further e-mail was sent. Where findings from a trial have been reported across multiple papers, we selected the paper in which mindfulness data are reported or the study with the larger sample size. Reference lists of the final set of papers were searched manually to identify additional papers not identified in the original search.

### Inclusion and Exclusion Criteria

We included studies that (a) were randomized controlled trials, (b) recruited adults (aged 18 years or over), (c) compared an MBI to an active control condition (face-to-face or non-face-to-face condition) that did not include explicit mindfulness training, where “active control” is defined in line with the Cochrane Handbook 5.1 as a different kind of therapy or treatment (Higgins & Green (2011), (d) included an empirically supported measure of mindfulness, and (e) evaluated MBSR, MBCT, or a well-established variant (Breathworks, mindfulness-based living course, mindfulness-based childbirth and parenting, and mindfulness-based relapse prevention).

We excluded studies that were not reported in the English language and evaluated an MBI that (a) was not delivered in person (self-help or online MBIs), (b) was not delivered in a group format, (c) had fewer than eight sessions or less than 12 hr of face-to-face contact with a trained MBI facilitator, or (d) compared the MBI only to an inactive control condition where *inactive control* is defined in line with the Cochrane Handbook 5.1 as including a placebo, no treatment, standard care, or a waiting list control. Group format was required because the well-established MBIs we tested were designed for group delivery and the evidence base supporting them is based almost entirely on this format.

### Data Extraction and Analysis

For each condition, baseline and postintervention means, standard deviations, and number of participants for measures of mindfulness were extracted and entered into Comprehensive Meta-Analysis (Version 3.0; Borenstein, Hedges, Higgins, & Rothstein, 2013). Study characteristics for the moderator analyses described later also were entered. All data were extracted by Jenny Gu and any uncertainties or queries that arose were resolved in discussion with the other authors.

Pre–post between-Group Hedges’ *g* effect sizes, 95% confidence intervals (CIs), and *z* and *p* values were computed. The pre–post between-groups effect size reflects the difference between pre–post change in the MBI group and pre–post change in the active control. By convention, a small effect size is considered to be 0.2, a medium effect size is 0.5, and a large effect size is 0.8 (Cohen, 1988). The overall Hedges’ *g* effect size was computed using a random effects model because of differences between included studies (e.g., in the mindfulness measure used, control group). Under a random effects model, the pooled effect size is the weighted average of individual Hedges’ *g* effect sizes, with each study weighted by the inverse of its variance (sum of within-study and between-study variance).

Data were extracted and meta-analyses were performed for six outcomes: the total mindfulness score from any empirically supported measure of mindfulness, facet scores for observing, describing, acting with awareness, and nonjudging from the KIMS or FFMQ, and the nonreactivity facet from the FFMQ. Where standard deviations were not provided, they were calculated from standard errors and confidence intervals.

Forest plots of pre–post between-groups effect sizes were produced for each of the six outcomes and for moderator analyses. Heterogeneity of effect sizes was assessed using the chi-square statistic (Cochrane’s *Q*) and *I*^2^ index. A significant *Q* value indicates heterogeneity of effect sizes. *I*^2^ indicates the percentage of variance in effect sizes attributable to true, between-study heterogeneity rather than sampling error or chance. *I*^2^ values of around 25%, 50%, and 75% can be considered as indicating low, moderate, and high heterogeneity, respectively (Higgins & Thompson, 2002).

Moderator analyses were planned for (a) which mindfulness measure was used (e.g., FFMQ, MAAS, KIMS), (b) type of control condition (CBT or CBT-based, medication, other), (c) whether the control intervention was matched to the MBI for same/greater amount of face-to-face contact and number of sessions, and (d) whether formal fidelity checks for the MBI were reported. Subgroup effect sizes are reported when the moderator analysis is significant.

To address publication bias, a funnel plot was produced and the trim and fill method was used. Rosenthal’s (1979) Fail-Safe *N* and Begg and Mazumdar’s (1994) rank correlation test were also computed for the analysis of mindfulness total scores. Funnel plots display study effect sizes against their standard errors; points evenly distributed around the mean effect size (represented as a vertical line) and forming a symmetrical inverted funnel shape indicate that publication bias is unlikely. Publication bias is suggested if the funnel shape is distorted such that there is a disproportionate number of studies with larger standard errors (generally studies with smaller samples) on the side of the mean favoring the intervention condition. This would suggest that smaller studies are more likely to be published if they found larger effects and that studies with effects favoring the control condition may be missing from the published literature. The trim-and-fill method provides an estimate of the number of missing studies and an adjusted overall mean effect size. Rosenthal’s Fail-Safe *N* estimates the number of unpublished studies with similar sample sizes and with effect sizes of zero that would be needed to reduce the mean effect size to nonsignificance. Effect sizes can be considered robust if the required number of unpublished studies is greater than or equal to *5k* + 10, where *k* is the number of studies in a meta-analysis (Rosenberg, 2005). Begg and Mazumdar’s rank correlation test examines the rank correlation between standardized effect sizes and their standard errors using Kendall’s tau. Publication bias would be indicated by a significant correlation between effect size and standard error, with smaller studies (with larger standard errors) associated with larger effect sizes.

The Cochrane Collaboration’s risk of bias tool (Higgins et al., 2011) was used to assess the risk of bias in each study (low, unclear, or high risk of bias) using the following seven criteria: adequacy of random sequence generation, concealment of the allocated intervention from participants and investigators, blinding of participants and personnel to the intervention allocation, blinding of outcome assessors to intervention allocation, completeness of outcome data (whether attrition, exclusions, and missing data were adequately addressed), evidence of selective outcome reporting, and other sources of bias. Risk of bias was not assessed for one unpublished study (Simshauser, Luking, Kaube, Schultz, & Schmidt, in press; ClinicalTrials.gov: NCT00826475). A total quality score was computed for each study, with 1 point awarded for low risk of bias and 0 points awarded for high or unclear risk for each of the seven criteria. Quality scores ranged from 0 to 7. Correlations (Pearson’s *r*) between quality scores and effect sizes for total mindfulness scores were computed.

## Results

### Search Results

**Figure 1 fig1:**
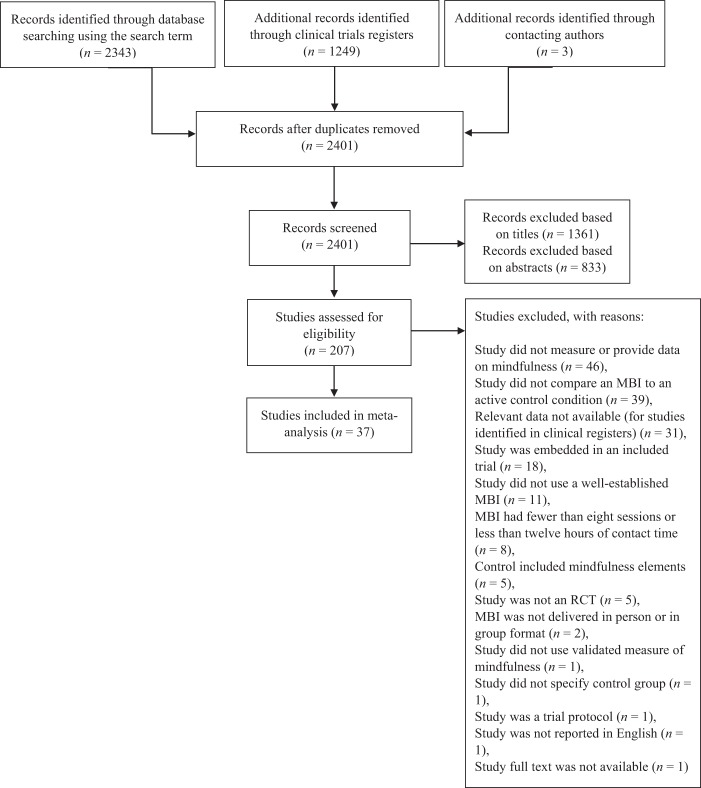
Flow diagram of the study selection process.

Figure 1 shows the study selection process. Searching of databases using the terms described earlier yielded 2,343 records. An additional 1,249 papers were identified through clinical trials registers, and 3 were identified by contacting authors. After removing duplicates, 2,401 records remained. Of these, 1,361 were excluded based on the title and 833 were excluded based on the abstract. The full texts of the remaining 207 papers were examined and inclusion and exclusion criteria applied. After exclusions for the reasons detailed in Figure 1, 37 studies remained for inclusion in the meta-analysis.[Fig-anchor fig1]

### Study Characteristics

Characteristics of the 37 included studies are displayed in Table 2. All measured mindfulness pre- and posttreatment in a randomized trial comparing an MBI to an active control. The most commonly used mindfulness measure was the FFMQ (*k* = 19), followed by the MAAS (*k* = 9), FMI (*k* = 3), KIMS (*k* = 3), CAMS–R (*k* = 2), and the Toronto Mindfulness Scale (TMS; Lau et al., 2006; *k* = 1). The total number of participants was 4,108 at baseline; 2,056 of these were randomized to MBIs and 2,052 to control conditions. Mean age ranged from 29 to 75 years. In most studies, participants were experiencing a current episode of a diagnosed mental health disorder (*k* = 10) or a diagnosed physical health condition (*k* = 11). Other studies included participants who were currently in remission from a diagnosed mental health disorder (*k* = 6) or community samples (*k* = 3). Seven studies recruited participants who did not clearly fall under these subgroups (e.g., caregivers scoring above a threshold on a measure of strain, current cigarette smokers).

Almost all studies examined MBSR (*k* = 21) or MBCT (*k* = 15). One study examined MBRP (Witkiewitz et al., 2014). Most studies (*k* = 24) used modified protocols of MBCT or MBSR. Modifications included adaptations for the population, providing more than eight sessions, shortening the duration of sessions, and omitting the all-day retreat. The number of weekly sessions for MBIs ranged from eight to 16 and the total number of in-session hours ranged from 12 to 30. Of the 37 included studies, 18 used active control interventions matched for the same or greater amount of face-to-face contact time and number of sessions as the MBI. There was a range of active control conditions, including exercise programs, medication, group health enhancement or education programs, group CBT, and self-help materials.

### Meta-Analysis Results for Mindfulness Total Scores

**Figure 2 fig2:**
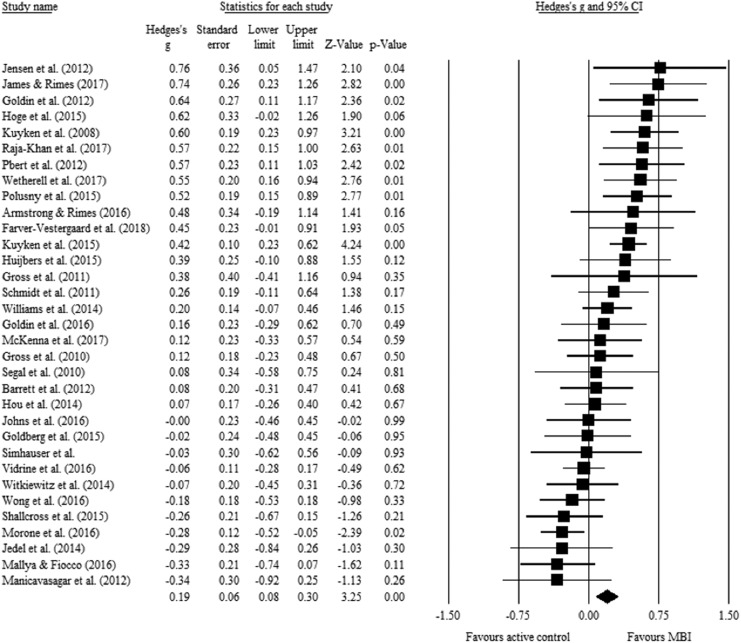
Forest plot of the effect of mindfulnessbased intervention (MBIs) compared to active control conditions on pre–post total mindfulness scores. Standardized mean difference values shown are Hedges’ *g* effect sizes.

Mean effect sizes (weighted by sample size) for mindfulness total scores are shown in Table 3. A random effects model on the 33 studies that reported mindfulness total scores (see Figure 2) showed a pre–post between-groups difference in favor of the MBI over the active control condition. The effect size was small (Hedges *g* = 0.19, 95% CI [0.08, 0.30]) and statistically significant (*z* = 3.25, *k* = 33, *p* < .001). Heterogeneity was significant and moderate-high, *Q*(32) = 82.86, *p* < .001; *I*^2^ = 61.38%. Moderator analyses were conducted to examine potential sources of heterogeneity.[Table-anchor tbl2][Table-anchor tbl3][Fig-anchor fig2]

### Moderator Analyses

Moderator analyses were conducted only for total mindfulness scores because fewer studies reported facet-level scores. Mean effect sizes for mindfulness total scores for each questionnaire were shown in Table 3; mean effect sizes for the other potential moderators are shown in Table 4. Effect sizes for individual studies reporting total scores, classified by the four potential moderators, are shown in Table 5. Forest plots are shown in the Supplemental Figures S1–S9 in the online supplemental material.[Table-anchor tbl4][Table-anchor tbl5]

#### Mindfulness measure

Difference between MBIs and active controls in pre–post change in mindfulness scores varied significantly depending on which measure of mindfulness was used, *Q*(4) = 11.35, *p* = .02. MBIs showed significantly greater pre–post change in mindfulness scores than were seen in the active control conditions when mindfulness was measured with the FFMQ, with a small-medium effect size (Hedges *g* = 0.25, 95% confidence interval (CI) [0.10, 0.40], *z* = 3.34, *k* = 15, *p* < .001), and when mindfulness was measured with the CAMS–R, with a medium effect size (Hedges *g* = 0.52, 95% CI [0.17, 0.87], *z* = 2.89, *k* = 2, *p* = .004). However, the between-groups pre–post difference in mindfulness was nonsignificant when measured using the MAAS (Hedges *g* = −0.06, 95% CI [−0.25, 0.14], *z* = −0.59, *k* = 9, *p* = .55), KIMS (Hedges *g* = 0.36, 95% CI [−0.15, 0.88], *z* = 1.39, *k* = 3, *p* = .17), and FMI (Hedges *g* = 0.08, 95% CI [−0.17, 0.32], *z* = 0.64, *k* = 3, *p* = .53). The TMS was used in only one study (Raja-Khan et al., 2017) and was excluded from this analysis.

#### Type of control condition

Moderator analysis showed a significant difference in pre–post change in mindfulness between studies using cognitive–behavioral, medication, or other interventions as the active control condition, *Q*(2) = 8.75, *p* = .01. As expected, MBIs showed significantly greater pre–post change in mindfulness scores when compared to medication, with a small-medium effect size (Hedges *g* = 0.43, 95% CI [0.28, 0.59], *z* = 5.45, *k* = 5, *p* < .001) and when compared to other psychosocial but non-CBT conditions, with a small effect size (Hedges *g* = 0.18, 95% CI [0.03, 0.34], *z* = 2.28, *k* = 20, *p* = .02). When compared to CBT or CBT-based active control conditions, MBIs did not significantly differ in pre–post change in mindfulness (Hedges *g* = 0.08, 95% CI [−0.12, 0.28], *z* = 0.82, *k* = 8, *p* = .42).

#### Matching for number and duration of sessions

Pharmacotherapy typically does not involve lengthy sessions and is not designed to match the session time of psychosocial interventions. Therefore, this analysis excluded the five studies for which medication was the control condition, leaving 28 studies. To provide a rigorous test of matched session time as a moderating variable, studies in which session number and duration for the active control group equaled or exceeded the MBI were coded as matched; studies in which the active control had less session time than the MBI were coded as unmatched. Moderator analysis showed a significant difference in pre–post change in mindfulness between studies which were matched or unmatched for number and duration of sessions, *Q*(1) = 7.83, *p* = .005. MBIs showed significantly greater pre–post change in mindfulness scores when compared to unmatched control conditions, with a small-medium effect size (Hedges *g* = 0.34, 95% CI [0.17, 0.51], *z* = 3.90, *k* = 12, *p* < .001), but not when compared to matched active control conditions (Hedges *g* = 0.02, 95% CI [−0.16, 0.16], *z* = 0.33, *k* = 16, *p* = .74).

#### Fidelity checking for the MBI

Moderator analysis showed that the difference in pre–post mindfulness between studies reporting and not reporting formal fidelity checks was not significant, *Q*(1) = 0.51, *p* = .48.

### Meta-Analysis Results for Mindfulness Facet Scores (FFMQ/KIMS)

Random effects models were examined for each of the five facets of mindfulness as measured by the KIMS or FFMQ. Mean effect sizes for mindfulness facet scores are shown in Table 3.

#### Observing

The between-groups difference for pre–post observing was significant and favored the MBI, with a small-medium effect size (Hedges *g* = 0.24, 95% CI [0.09, 0.38], *z* = 3.18, *k* = 17, *p* = .001). Heterogeneity was significant and moderate-high, *Q*(16) = 43.84, *p* < .001; *I*^2^ = 63.50%.

#### Describing

The between-groups difference for pre–post describing was nonsignificant (Hedges *g* = 0.05, 95% CI [−0.05, 0.15], *z* = 1.00, *k* = 16, *p* = .32). Heterogeneity was nonsignificant, *Q*(15) = 18.91, *p* = .22; *I*^2^ = 20.68%.

#### Acting with awareness

The between-groups difference for pre–post acting with awareness was nonsignificant (Hedges *g* = 0.02, 95% CI [−0.08, 0.12], *z* = 0.43, *k* = 17, *p* = .67). Heterogeneity was nonsignificant, *Q*(16) = 20.93, *p* = .18; *I*^2^ = 23.56%.

#### Nonjudging

The between-groups difference for pre–post nonjudging was significant and favored the MBI. Effect size was small (Hedges *g* = 0.14, 95% CI [0.05, 0.23], *z* = 3.10, *k* = 17, *p* = .002). Heterogeneity was nonsignificant, *Q*(16) = 17.72, *p* = .34; *I*^2^ = 9.72%.

#### Nonreactivity

The between-groups difference for pre–post nonreactivity was significant and favored the MBI with a small-medium effect size (Hedges *g* = 0.23, 95% CI [0.08, 0.39], *z* = 2.91, *k* = 15, *p* = .004). Heterogeneity was significant and moderate-high, *Q*(14) = 35.98, *p* = .001; *I*^2^ = 61.09%.

### Publication Bias

**Figure 3 fig3:**
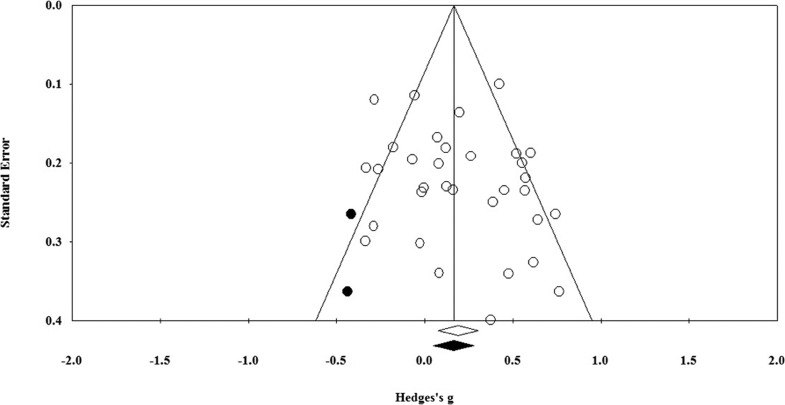
Funnel plot of effect sizes by standard error for pre–post total mindfulness scores.

The Trim and Fill method indicates that two studies would need to fall on the left of the mean effect size to make the funnel plot symmetrical (see Figure 3). In a random-effects model, the new imputed mean effect size would be Hedges *g* = 0.17, 95% CI [0.05, 0.28]. Rosenthal’s Fail-Safe *N* analysis found that an additional 193 unpublished studies with effect sizes of zero would be needed to reduce the mean effect size for mindfulness total scores to nonsignificance. This figure is greater than 175 (*5k* + 10, where *k* = 33), which suggests that effect sizes can be considered robust (Rosenberg, 2005). Kendall’s tau was small and nonsignificant (Kendall’s τ = .09, *k* = 33, *p* = .439). Taken together, these do not indicate the presence of publication bias.[Fig-anchor fig3]

### Relationship Between Study Quality and Effect Size for Mindfulness Total Scores

Total quality scores for each study, based on risk of bias, are shown in Table 2. Scores for each criterion are shown in Supplemental Table S1 and Supplemental Figure S10 (in the online supplementary materials). Supplemental Figure S11 (in the online supplementary materials) displays percentages of studies with low, unclear, and high risk of bias for each criterion. Most studies had a low risk of bias for all criteria apart from the ‘selective outcome reporting’ criterion, for which most had an unclear risk of bias.

The correlation between study quality scores and pre–post between-groups effect sizes for mindfulness total scores was nonsignificant, *r*(30) = −.02, *p* = .935. This suggests that greater risk of bias, indicated by lower quality scores, is not associated with larger effects.

## Discussion

The psychometric properties of mindfulness questionnaires are generally well supported; however, studies showing that self-reported mindfulness sometimes improves in interventions with no explicit mindfulness training have raised a question about their differential sensitivity to change with treatment (Goldberg et al., 2016; Visted et al., 2015).We synthesized 37 studies to examine whether interventions explicitly designed to teach mindfulness lead to greater changes in self-reported mindfulness skills than comparison interventions with no explicit mindfulness training. When all studies were included in the analysis, results were as expected. That is, participants in MBIs showed significantly greater pre–post improvements in mindfulness scores than were seen in active control conditions with no explicit mindfulness elements. The mean effect size was small. The trim-and-fill method and Rosenthal’s fail-safe *N* suggested that publication bias was not a concern. However, the overall finding was moderated by several variables, including which mindfulness questionnaire was used, the type of treatment offered in the control condition, and whether the MBI and control condition were matched for session time. The implications of each of the moderator analyses are discussed in turn.

### Which Questionnaire Was Used

The FFMQ and CAMS–R showed significant differential sensitivity to change with treatment but the other measures did not. For the KIMS, mean effect size was larger than for the FFMQ but was not statistically significant, perhaps because the KIMS was used in only three studies. The TMS also showed a medium effect size but was used in only one study. In contrast, mean effect sizes for the MAAS and FMI were near zero. Of the nine effect sizes for the MAAS, one was large whereas eight were close to zero or in the unexpected direction. Of the three effect sizes for the FMI, two were near zero and one was small.

It is unclear why some of the mindfulness questionnaires showed better differential sensitivity than others. Facet-level analyses showed significant effect sizes for observing, nonjudging, and nonreactivity but not for describing or acting with awareness. It is possible that the multifaceted instruments more fully represent the breadth of the mindfulness construct, and therefore are better able to capture skills that change more with mindfulness training than with other interventions. This could explain the larger effect sizes for the FFMQ and KIMS. The CAMS–R, though providing only a total score, also includes considerable breadth of content (present-moment focus, awareness of thoughts and feelings, nonjudgment, acceptance). In contrast, the MAAS, which had a mean effect size near zero, is more narrowly focused on general attentiveness. The FMI includes content related to awareness, nonjudging, and nonreactivity, but also includes more general items that may change with other interventions, such as impatience, staying calm under stress, considering different perspectives, and general self-acceptance (rather than acceptance of thoughts and feelings). This more general content may explain why the FMI showed similar increases in MBIs and other psychosocial interventions.

### Type of Active Control Condition

Mindfulness skills increased significantly more in MBIs than with medication. This was predicted because medication is not expected to teach mindfulness skills. However, pre–post change in mindfulness did not differ significantly between MBIs and CBT-based controls. This finding could be explained in several ways. The MBIs may have failed to teach mindfulness adequately, or the questionnaires may be sensitive to changes in distress, which improves in wide range of interventions. Alternatively, the questionnaires may measure mindfulness-related skills that are taught explicitly in MBIs and cultivated implicitly in CBT. We argue that the latter explanation is the most likely, for several reasons.

First, although the studies provide little information about the adequacy of the mindfulness teaching, it seems unlikely that they failed to teach mindfulness skills. All used MBIs with strong empirical support that are consistent with the defining features of MBIs as described by Crane et al. (2017). Second, medication is expected to improve distress but does not directly teach skills; thus, the significant effect size for the comparison of MBIs to medication (g = .43) suggests that the mindfulness questionnaires measure something that changes with mindfulness training but not with medication. Third, CBT cultivates decentering (Farb et al., 2018; Fresco et al., 2007), which is strongly correlated with self-reported mindfulness (Carmody et al., 2009). This suggests that any intervention that increases decentering is likely to lead to increases in self-reported mindfulness skills, even if decentering is taught using nonmindfulness-based methods.

Finally, a randomized trial comparing MBSR and CBT for social anxiety (Goldin et al., 2016, included in this meta-analysis) showed that changes can occur in psychological process that are not explicitly targeted by the treatment. The study found that MBSR and CBT were equally effective in reducing social anxiety and more effective than a waitlist control. Measures of potential mechanisms of action for both interventions were included.

Unexpectedly, both treatments led to significant and similar improvements in most of the potential mechanisms, including mindfulness skills, cognitive distortions, and cognitive reappraisal. That is, CBT led to increased mindfulness despite the absence of explicit mindfulness training; similarly, MBSR led to changes in cognitive reappraisal and cognitive distortions, despite the absence of explicit training in cognitive restructuring. Although this could be interpreted as a lack of differential sensitivity for the FFMQ, the Emotion Regulation Questionnaire (Gross & John, 2003), and the Cognitive Distortions Questionnaire (Morrison et al., 2015), Goldin et al. (2016) concluded that CBT and MBSR share more underlying psychological processes than is commonly recognized and that, in both interventions, some of these processes may change without explicit training.

### Matching of Session Time

When the MBIs and the active psychosocial controls were matched for session time, there was no significant difference in pre–post change in mindfulness scores, suggesting a lack of differential sensitivity to change with treatment. This might suggest that the questionnaires are sensitive to changes that occur in a variety of interventions, such as reductions in psychological symptoms. It is also possible that when CBT-based and other psychosocial interventions are matched for session time with the MBI, the cultivation of decentering and other mindfulness-related skills approximates the cultivation of mindfulness in the MBIs, leading to similar increases in self-reported mindfulness. Only additional research can show whether either of these explanations is correct. Studies are needed to clarify the conditions that lead to acquisition of mindfulness skills in evidence-based MBIs and other interventions. Dismantling studies that allow testing of the effects of specific elements of MBIs and other interventions on self-reported mindfulness skills may be informative. Studies could also examine whether revisions to mindfulness questionnaires would increase their specificity to increases in mindfulness skills.

### Presence or Absence of a Fidelity Check

The only nonsignificant moderation analysis compared MBIs with and without a fidelity check. We argued earlier that the examination of our central research question is less ambiguous when the MBIs can be expected to teach mindfulness skills effectively. Accordingly, we included only studies using evidence-based protocols that meet the definition of MBI proposed by Crane et al. (2017). Even with this restriction, it is possible that some studies implemented the MBI more skillfully than others. Because many studies do not include or report the results of fidelity checks, we had no direct information about how competently the MBIs were implemented and relied on presence or absence of a fidelity check as a proxy for adherence to the protocol. The nonsignificant moderation analysis may mean that presence of a fidelity check does not reflect competence in intervention delivery, or that competence in intervention delivery was not related to the cultivation of mindfulness skills, perhaps because of the restriction of range in therapists’ competence.

### Limitations

The included studies yielded a wide range of effect sizes and the moderating variables seem to account for only some of this heterogeneity. For example, when considering only studies using the FFMQ, effect sizes ranged from −.26 to .74 (see Table 5). For FFMQ studies that were matched for session time and included a fidelity check, effect sizes ranged from −.26 to .62. Variables other than the moderators we tested may be important in accounting for some of this heterogeneity. Additional work is necessary to identify factors related to differences between MBIs and other treatments in the cultivation of mindfulness skills.

The inclusion of only MBSR, MBCT, and evidence-based variants that meet the definition of MBI proposed by Crane et al. (2017) may be a limitation, in that it omits single session mindfulness trainings, laboratory-based inductions, and other training with little empirical support. This decision was made to circumvent the difficulty in interpreting the findings when the MBI and the active control show similar increases in self-reported mindfulness. By including only well-established MBIs, we made it unlikely that an apparent lack of differential sensitivity of a mindfulness questionnaire could be attributed to poor teaching of mindfulness in the MBI. This leaves two other explanations, as noted earlier. First, the questionnaire may be sensitive to changes in a more general construct, such as distress, that improves with a variety of interventions. Second, the active control conditions may implicitly teach mindfulness-related skills. Our findings suggest that the second explanation is more likely, at least for some mindfulness questionnaires, because effect sizes were larger when comparing MBIs to medication controls, which reduce distress but are not expected to teach mindfulness skills, than to CBT or other psychosocial controls, which may implicitly teach skills related to mindfulness.

The number of studies available may be a limitation for the moderation analyses, which must be interpreted cautiously. Although three of the four moderation analyses were significant, they should be replicated when the number of available studies has grown. Moreover, we conducted only univariate moderation analyses despite the potential importance of combined effects of the proposed moderators. For example, it could be argued that the most stringent test of the differential sensitivity of mindfulness questionnaires would examine only studies that were matched for session time, included a fidelity check, and compared an MBI to a non-CBT and nonmedication control condition. Unfortunately, as shown in Table 5, there are only five such studies (three with the FFMQ, two with the MAAS). If we expand to include all types of comparison groups (and collapse across them), there are only nine studies (six with the FFMQ, three with the MAAS). Multivariate moderation analyses with such small cell sizes are likely to be misleading (Lipsey, 2003).

## Conclusions

Although findings provide partial support for the differential sensitivity of mindfulness questionnaires to change with treatment, this effect was not found when the MBI and control were matched for session time. Potential explanations for this were suggested, but further research is needed to clarify whether revisions of mindfulness questionnaires would increase their specificity to the changes that occur with mindfulness training, or whether both MBIs and other psychosocial interventions cultivate mindfulness skills. The findings suggest that for continued work in this area, multifaceted mindfulness measures, particularly the FFMQ, may be helpful in discriminating changes in mindfulness skills attributable to explicit mindfulness training from changes attributable to implicit cultivation of related skills or other factors.

## Supplementary Material

10.1037/pas0000744.supp

## Figures and Tables

**Table 1 tbl1:** Contemporary Psychological Descriptions of Mindfulness: What and How

Author(s)	What	How
Kabat-Zinn, 1994, 2003	Paying attention, or the awareness that arises through paying attention	On purpose, in the present moment, and nonjudgmentally; with an affectionate, compassionate quality, a sense of openhearted friendly presence and interest
Marlatt & Kristeller, 1999	Bringing one’s complete attention to present experiences	On a moment-to-moment basis, with an attitude of acceptance and loving kindness
Bishop et al., 2004	Self-regulation of attention so that it is maintained on immediate experience	With an orientation characterized by curiosity, openness, and acceptance
Germer, Siegel, & Fulton, 2005	Awareness of present experience	With acceptance: an extension of nonjudgment that adds a measure of kindness or friendliness
Linehan, 2015	The act of focusing the mind in the present moment	Without judgment or attachment, with openness to the fluidity of each moment

**Table 2 tbl2:**
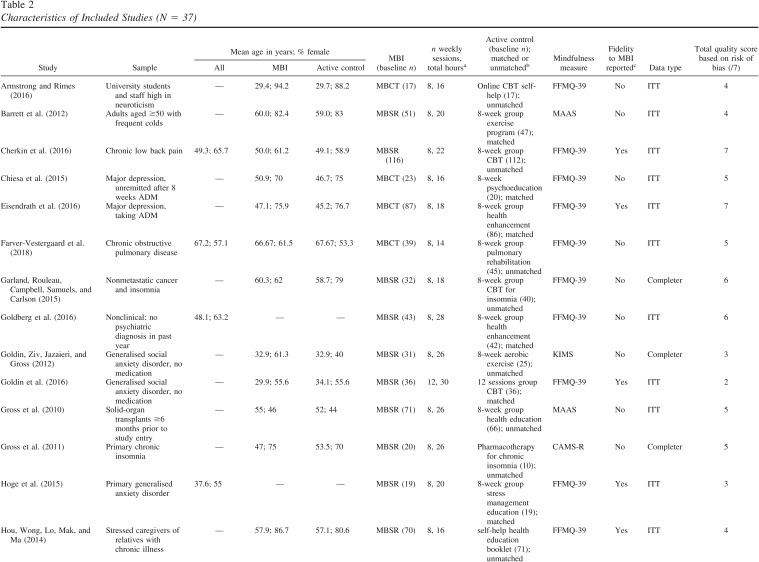
Characteristics of Included Studies (N = 37)

Study	Sample	Mean age in years; % female			MBI (baseline *n*)	*n* weekly sessions, total hours^a^	Active control (baseline *n*); matched or unmatched^b^	Mindfulness measure	Fidelity to MBI reported^c^	Data type	Total quality score based on risk of bias (/7)
		All	MBI	Active control							
Armstrong and Rimes (2016)	University students and staff high in neuroticism	—	29.4; 94.2	29.7; 88.2	MBCT (17)	8, 16	Online CBT self-help (17); unmatched	FFMQ-39	No	ITT	4
Barrett et al. (2012)	Adults aged ≥50 with frequent colds	—	60.0; 82.4	59.0; 83	MBSR (51)	8, 20	8-week group exercise program (47); matched	MAAS	No	ITT	4
Cherkin et al. (2016)	Chronic low back pain	49.3; 65.7	50.0; 61.2	49.1; 58.9	MBSR (116)	8, 22	8-week group CBT (112); unmatched	FFMQ-39	Yes	ITT	7
Chiesa et al. (2015)	Major depression, unremitted after 8 weeks ADM	—	50.9; 70	46.7; 75	MBCT (23)	8, 16	8-week psychoeducation (20); matched	FFMQ-39	No	ITT	5
Eisendrath et al. (2016)	Major depression, taking ADM	—	47.1; 75.9	45.2; 76.7	MBCT (87)	8, 18	8-week group health enhancement (86); matched	FFMQ-39	Yes	ITT	7
Farver-Vestergaard et al. (2018)	Chronic obstructive pulmonary disease	67.2; 57.1	66.67; 61.5	67.67; 53.3	MBCT (39)	8, 14	8-week group pulmonary rehabilitation (45); unmatched	FFMQ-39	No	ITT	5
Garland, Rouleau, Campbell, Samuels, and Carlson (2015)	Nonmetastatic cancer and insomnia	—	60.3; 62	58.7; 79	MBSR (32)	8, 18	8-week group CBT for insomnia (40); unmatched	FFMQ-39	No	Completer	6
Goldberg et al. (2016)	Nonclinical: no psychiatric diagnosis in past year	48.1; 63.2	—	—	MBSR (43)	8, 28	8-week group health enhancement (42); matched	FFMQ-39	No	ITT	6
Goldin, Ziv, Jazaieri, and Gross (2012)	Generalised social anxiety disorder, no medication	—	32.9; 61.3	32.9; 40	MBSR (31)	8, 26	8-week aerobic exercise (25); unmatched	KIMS	No	Completer	3
Goldin et al. (2016)	Generalised social anxiety disorder, no medication	—	29.9; 55.6	34.1; 55.6	MBSR (36)	12, 30	12 sessions group CBT (36); matched	FFMQ-39	Yes	ITT	2
Gross et al. (2010)	Solid-organ transplants ≥6 months prior to study entry	—	55; 46	52; 44	MBSR (71)	8, 26	8-week group health education (66); unmatched	MAAS	No	ITT	5
Gross et al. (2011)	Primary chronic insomnia	—	47; 75	53.5; 70	MBSR (20)	8, 26	Pharmacotherapy for chronic insomnia (10); unmatched	CAMS-R	No	Completer	5
Hoge et al. (2015)	Primary generalised anxiety disorder	37.6; 55	—	—	MBSR (19)	8, 20	8-week group stress management education (19); matched	FFMQ-39	Yes	ITT	3
Hou, Wong, Lo, Mak, and Ma (2014)	Stressed caregivers of relatives with chronic illness	—	57.9; 86.7	57.1; 80.6	MBSR (70)	8, 16	self-help health education booklet (71); unmatched	FFMQ-39	Yes	ITT	4
Huijbers et al. (2015)	≥3 prior depressive episodes, full or partial remission, taking ADM	—	51.9; 73	51.6; 71	MBCT (33)	8, 26	Maintenance ADM (35); unmatched	FFMQ-39	Yes	ITT	5
James and Rimes (2018)	University students high in perfectionism	—			MBCT (28)	8, 16	Self-help psychoeducation booklet (32); unmatched	FFMQ-39	No	ITT	5
Jedel et al. (2013)	Inactive ulcerative colitis	—	46.0; 44.4	39.7; 67.9	MBSR (27)	8, 20	8-week group mind/body medicine psychoeducation (28); unmatched	MAAS	No	ITT	6
Jensen, Vangkilde, Frokjaer, and Hasselbalch (2012)	Nonclinical: healthy students	range: 20–36; 66	—	—	MBSR (16)	8, 27	8-week group stress reduction course (16); unmatched	MAAS	No	ITT	4
Johns et al. (2016)	Nonmetastatic breast and colorectal cancer and persistent fatigue	—	56.9; 94.3	56.4; 86.1	MBSR (35)	8, 16	8-week group support and psychoeducation (36); matched	FFMQ-39	Yes	ITT	7
Kuyken et al. (2008)	≥3 prior depressive episodes, full or partial remission, taking ADM	—	49.0; 77	49.4; 76	MBCT (61)	8, 16	Maintenance ADM (62); unmatched	KIMS	Yes	ITT	6
Kuyken et al. (2015)	≥3 prior depressive episodes, full or partial remission, taking ADM	—	50; 71	49; 82	MBCT (212)	8, 18	Maintenance ADM (212); unmatched	FFMQ-39	Yes	ITT	6
Mallya and Fiocco (2016)	Healthy adults ≥60 years living independently in community	—	68.8; 77	69.7; 70	MBSR (57)	8, 20	8-week reading and relaxation group (40); matched	MAAS	Yes	ITT	4
Manicavasagar, Perich, and Parker (2012)	Major depression	—	47; 63	45; 65	MBCT (30)	8, 20	8-week group CBT (39); matched	MAAS	Yes	Completer	5
McKenna, Marks, Hallsworth, and Schaette (2017)	Adults with tinnitus and clinical levels of psychological distress	median (IQR): 50 (16); 45	median (IQR): 47 (17); 46	median (IQR): 53 (14); 44	MBCT (39)	8, 16	8-week group relaxation training (36); matched	MAAS	Yes	ITT	6
Morone et al. (2016)	≥65 years, chronic low back pain	—	75; 66.4	74; 66.2	MBSR (140)	8, 12	8-week group health education (142); matched	MAAS	No	ITT	7
Pbert et al. (2012)	Persistent asthma	—	51.9; 64.3	53.6; 70.7	MBSR (42)	8, 26	8-week group healthy living course (41); matched	FFMQ-39	No	ITT	5
Polusny et al. (2015)	Veterans with full or subthreshold PTSD	58.5; 16	57.6; 21	59.4; 10	MBSR (58)	8, 26 hours 30 mins	9-week present-centred group therapy (58); unmatched	FFMQ-39	Yes	ITT	7
Raja-Khan et al. (2017)	Women who are overweight or obese	44.5; 100	47.0; 100	42.2; 100	MBSR (42)	8, 26	8-week group health education course (44); matched	TMS	No	ITT	7
Schmidt et al. (2011)	Women with fibromyalgia	52.5; 100	53.4; 100	51.9; 100	MBSR (53)	8, 27	8-week group relaxation, stretching, education, support (59); unmatched	FMI	No	ITT	6
Segal et al. (2010)	≥2 prior depressive episodes, in remission	44; 63	44.8; 50	45.8; 71	MBCT (26)	8, 22	Maintenance ADM (28); unmatched	MAAS	Yes	ITT	5
Shallcross et al. (2015)	≥1 prior depressive episode, in remission	—	36.7; 76	33; 76	MBCT (46)	8, 20	Group health enhancement (46); matched	FFMQ-39	Yes	ITT	5
Simshäuser et al. (in press; ClinicalTrials.gov: NCT00826475)	Migraine with or without aura, ≥2 attacks per month	—	43.8; 90.3	43.9; 93.3	MBSR (31)	8, 26	3 sessions education and relaxation (30); unmatched	FMI	No	Completer	—
Vidrine et al. (2016)	Current cigarette smokers motivated to quit smoking	48.7; 54.9	48.4; 54.6	48.8; 54.2	MBCT (154)	8, 16	8-week group CBT (155); matched	KIMS	No	ITT	5
Wetherell et al. (2017)	Current depressive and/or anxiety disorder and current subjective aging-related neurocognitive problems	71.9; 73	70.4; 72	73.3; 73	MBSR (47)	8, 15	8 week group health education program (56); unmatched	CAMS-R	No	ITT	6
Williams et al. (2014)	≥3 prior episodes of MDD, in remission	43; 72	—	—	MBCT (108)	8, 16	8 week group cognitive psychoeducation (110); matched	FFMQ-39	Yes	ITT	7
Witkiewitz et al. (2014)	Women with history of illegal activity (drug use/possession, burglary, prostitution)	—	35.8; 100	32.4; 100	MBRP (55)	16 sessions in 8 weeks, 13 hours 20 mins	16 sessions over 8 weeks group relapse prevention (50); matched	FMI	No	ITT	4
Wong et al. (2016)	Generalised anxiety disorder	50; 79.1	50.4; 78.7	50.8; 78.7	MBCT (61)	8, 16	8 week group CBT-based psychoeducation (61); matched	FFMQ-39	Yes	ITT	4
*Note*. MBI = mindfulness-based intervention; MBCT = mindfulness-based cognitive therapy; MBSR = mindfulness based stress reduction; MAAS = Mindful Attention Awareness Scale; KIMS = Kentucky Inventory of Mindfulness Skills; CAMS–R = Cognitive Affective Mindfulness Scale—Revised; TMS = Toronto Mindfulness Scale; FMI = Freiburg Mindfulness Inventory; FFMQ = Five Facet Mindfulness Questionnaire; MBRP = mindfulness-based relapse prevention; ITT = intent to treat.											
^a^ The total number of hours in the MBI includes the retreat. If the length of the retreat was not mentioned, this was assumed to be six hours. ^b^ Whether the active control condition was matched or unmatched for same or greater amount of face-to-face contact time and number of sessions as the MBI. ^c^ Whether formal checks of fidelity to the MBI are reported in the paper.											

**Table 3 tbl3:** Mean Pre–Post Between-Group Effect Sizes for Total Mindfulness and Mindfulness Facet Scores

Mindfulness measure	*n*	Hedges’ *g*	95% CI	*z*	*p*
CAMS–R total	2	.52	[.17, .87]	2.89	.004
KIMS total	3	.36	[−.15, .88]	1.39	.17
FMI total	3	.08	[−.17, .32]	.64	.53
MAAS total	9	−.06	[−.25, .14]	−.59	.55
FFMQ total	15	.25	[.10, .40]	3.34	<.001
Observing	17	.24	[.09, .38]	3.18	.001
Describing	16	.05	[−.05, .15]	1.00	.32
Acting with awareness	17	.02	[−.08, .12]	.43	.67
Nonjudging	17	.14	[.05, .23]	3.10	.002
Nonreactivity	15	.23	[.08, .39]	2.91	.001
*Note*. CI = confidence interval; CAMS–R = Cognitive Affective Mindfulness Scale—Revised; KIMS = Kentucky Inventory of Mindfulness Skills; FMI = Freiburg Mindfulness Inventory; MAAS = Mindful Attention Awareness Scale; FFMQ = Five Facet Mindfulness Questionnaire.					

**Table 4 tbl4:** Mean Pre–Post Between-Group Effect Sizes for Total Mindfulness Scores as a Function of Other Potential Moderating Variables

Potential moderator	*n*	Hedges’ *g*	95% CI	z	*p*
Session time and structure					
Matched	16	.02	[−.16, .16]	.33	.74
Unmatched	12	.34	[.17, .51]	3.90	<.001
Fidelity reported					
Yes	15	.15	[−.01, .31]	1.80	.07
No	18	.23	[.07, .40]	2.73	.006
Type of control condition					
CBT/CBT-based	8	.08	[−.12, .28]	.82	.42
Medication	5	.43	[.28, .59]	5.45	<.001
Other	20	.18	[.03, .34]	2.28	.02
*Note*. CI = confidence interval; CBT = cognitive-based therapy.					

**Table 5 tbl5:** Effect Sizes for Mindfulness Total Scores for Individual Studies, Categorized by Type of Active Control Group, Mindfulness Measure Used, Matching for Session Time, and Fidelity Check for the MBI

Comparison	FFMQ		KIMS		MAAS		FMI		CAMS–R		TMS^a^	
	First author	*g*	First author	*g*	First author	*g*	First author	*g*	First author	*g*	First author	*g*
MBI vs CBT-based	James	.74	Vidrine	**−.06**	Manicavasagar	***−.34***	Witkiewitz	**−.07**				
	Armstrong	.48										
	Williams	***.20***										
	Goldin (16)	***.16***										
	Wong	***−.18***										
MBI vs medication	Kuyken (15)	*.42*			Segal	*.08*			Gross (11)	.38		
	Huijbers	*.39*	Kuyken (08)	*.60*								
MBI vs other	Hoge	***.62***	Goldin (12)	.64	Jensen	.76	Schmidt	.26	Wetherell	.55	Raja-Khan	**.57**
	Pbert	**.57**			Gross (10)	.12						
	Polusny	*.52*			McKenna	***.12***						
	Farver-Vestergaard	.45			Barrett	**.08**	Simshauser	−.03				
	Hou	*.07*			Morone	**−.28**						
	Johns	***.00***			Jedel	−.29						
	Goldberg	**−.02**			Mallya	***−.33***						
	Shallcross	***−.26***										
*Note*. CI = confidence interval; FFMQ = Five Facet Mindfulness Questionnaire; KIMS = Kentucky Inventory of Mindfulness Skills; MAAS = Mindful Attention Awareness Scale; FMI = Freiburg Mindfulness Inventory; CAMS–R = Cognitive Affective Mindfulness Scale—Revised; TMS = Toronto Mindfulness Scale. Values in bold are from studies in which the mindfulness-based intervention (MBI) and active control were matched for session time. Values in italics are from studies that included a fidelity check of the MBI.												
^a^ Only one study used the TMS and this was excluded from the mindfulness measure moderator analysis.												

## References

[c1] Armstrong L., & Rimes K. A. (2016). Mindfulness-based cognitive therapy for neuroticism (stress vulnerability): A pilot randomized study. Behavior Therapy, 47, 287–298. 10.1016/j.beth.2015.12.005 27157024

[c2] Baer R. A. (2011). Measuring mindfulness. Contemporary Buddhism, 12, 241–261. 10.1080/14639947.2011.564842

[c3] Baer R. A., Smith G. T., & Allen K. B. (2004). Assessment of mindfulness by self-report: The Kentucky inventory of mindfulness skills. Assessment, 11, 191–206. 10.1177/1073191104268029 15358875

[c4] Baer R. A., Smith G. T., Hopkins J., Krietemeyer J., & Toney L. (2006). Using self-report assessment methods to explore facets of mindfulness. Assessment, 13, 27–45. 10.1177/1073191105283504 16443717

[c5] Baer R. A., Smith G. T., Lykins E., Button D., Krietemeyer J., Sauer S., . . . Williams M. G. (2008). Construct validity of the five facet mindfulness questionnaire in meditating and nonmeditating samples. Assessment, 15, 329–342. 10.1177/1073191107313003 18310597

[c6] Barrett B., Hayney M. S., Muller D., Rakel D., Ward A., Obasi C. N., . . . Coe C. L. (2012). Meditation or exercise for preventing acute respiratory infection: A randomized controlled trial. Annals of Family Medicine, 10, 337–346. 10.1370/afm.1376 22778122 PMC3392293

[c7] Begg C. B., & Mazumdar M. (1994). Operating characteristics of a rank correlation test for publication bias. Biometrics, 50, 1088–1101. 10.2307/2533446 7786990

[c81] Bishop S., Lau M., Shapiro S., Carlson L., Anderson N., Carmody J., . . . Devins G. (2004). Mindfulness: A proposed operational definition. Clinical Psychology: Science and Practice, 11, 230–241.

[c8] Borenstein M., Hedges L., Higgins J., & Rothstein H. (2013). Comprehensive meta-analysis (Version 3). Englewood, NJ: Biostat.

[c9] Brown K. W., & Ryan R. M. (2003). The benefits of being present: Mindfulness and its role in psychological well-being. Journal of Personality and Social Psychology, 84, 822–848. 10.1037/0022-3514.84.4.822 12703651

[c10] Buchheld N., Grossman P., & Walach H. (2001). Measuring mindfulness in insight meditation (Vipassana) and meditation-based psychotherapy: The development of the Freiburg Mindfulness Inventory (FMI). Journal of Meditation and Meditation Research, 1, 11–34.

[c11] Carmody J., Baer R. A., Lykins E. L. B., & Olendzki N. (2009). An empirical study of the mechanisms of mindfulness in a mindfulness-based stress reduction program. Journal of Clinical Psychology, 65, 613–626. 10.1002/jclp.20579 19267330

[c13] Chen S.-Y., & Zhou R.-L. (2014). Validation of a Chinese version of the Freiburg Mindfulness Inventory–Short version. Mindfulness, 5, 529–535. 10.1007/s12671-013-0208-8

[c14] Cherkin D. C., Sherman K. J., Balderson B. H., Cook A. J., Anderson M. L., Hawkes R. J., . . . Turner J. A. (2016). Effect of mindfulness-based stress reduction vs cognitive behavioral therapy or usual care on back pain and functional limitations in adults with chronic low back pain: A randomized clinical trial. Journal of the American Medical Association, 315, 1240–1249. 27002445 10.1001/jama.2016.2323 PMC4914381

[c15] Chiesa A., Castagner V., Andrisano C., Serretti A., Mandelli L., Porcelli S., & Giommi F. (2015). Mindfulness-based cognitive therapy vs. psycho-education for patients with major depression who did not achieve remission following antidepressant treatment. Psychiatry Research, 226, 474–483. 10.1016/j.psychres.2015.02.003 25744325

[c16] Christopher M., Neuser N., Michael P., & Baitmangalkar A. (2012). Exploring the psychometric properties of the Five Facet Mindfulness Questionnaire. Mindfulness, 3, 124–131. 10.1007/s12671-011-0086-x

[c17] Cohen J. (1988). Statistical power analysis for the behavioral sciences (2nd ed.). Hillsdale, NJ: Erlbaum.

[c18] Crane R. S., Brewer J., Feldman C., Kabat-Zinn J., Santorelli S., Williams J. M. G., & Kuyken W. (2017). What defines mindfulness-based programs? The warp and the weft. Psychological Medicine, 47, 990–999. 10.1017/S0033291716003317 28031068

[c19] Curtiss J., & Klemanski D. (2014). Factor analysis of the Five Facet Mindfulness Questionnaire in a heterogeneous clinical sample. Journal of Psychopathology and Behavioral Assessment, 36, 683–694. 10.1007/s10862-014-9429-y

[c20] Eisendrath S. J., Gillung E., Delucchi K. L., Segal Z. V., Nelson J. C., McInnes L. A., . . . Feldman M. D. (2016). A randomized controlled trial of mindfulness-based cognitive therapy for treatment-resistant depression. Psychotherapy and Psychosomatics, 85, 99–110. 10.1159/000442260 26808973 PMC4756643

[c21] Farb N., Anderson A., Ravindran A., Hawley L., Irving J., Mancuso E., . . . Segal Z. V. (2018). Prevention of relapse/recurrence in major depressive disorder with either mindfulness-based cognitive therapy or cognitive therapy. Journal of Consulting and Clinical Psychology, 86, 200–204. 10.1037/ccp0000266 29265831

[c22] Farver-Vestergaard I., O’Toole M. S., O’Connor M., Løkke A., Bendstrup E., Basdeo S. A., . . . Zachariae R. (2018). Mindfulness-based cognitive therapy in COPD: A cluster randomised controlled trial. The European Respiratory Journal, 51, 1702082 10.1183/13993003.02082-2017 29386337

[c23] Fresco D. M., Moore M. T., van Dulmen M. H., Segal Z. V., Ma S. H., Teasdale J. D., & Williams J. M. G. (2007). Initial psychometric properties of the experiences questionnaire: Validation of a self-report measure of decentering. Behavior Therapy, 38, 234–246. 10.1016/j.beth.2006.08.003 17697849

[c24] Fresco D. M., Segal Z. V., Buis T., & Kennedy S. (2007). Relationship of posttreatment decentering and cognitive reactivity to relapse in major depression. Journal of Consulting and Clinical Psychology, 75, 447–455. 10.1037/0022-006X.75.3.447 17563161

[c25] Garland S. N., Rouleau C. R., Campbell T., Samuels C., & Carlson L. E. (2015). The comparative impact of mindfulness-based cancer recovery (MBCR) and cognitive behavior therapy for insomnia (CBT-I) on sleep and mindfulness in cancer patients. Explore, 11, 445–454. 10.1016/j.explore.2015.08.004 26386748

[c83] Germer K., Siegel R., & Fulton P. (Eds.). (2005). Mindfulness and psychotherapy. New York, NY: Guilford.

[c26] Goldberg S. B., Wielgosz J., Dahl C., Schuyler B., MacCoon D. S., Rosenkranz M., . . . Davidson R. J. (2016). Does the Five Facet Mindfulness Questionnaire measure what we think it does? Construct validity evidence from an active controlled randomized clinical trial. Psychological Assessment, 28, 1009–1014. 10.1037/pas0000233 26460893 PMC4829487

[c27] Goldin P. R., Morrison A., Jazaieri H., Brozovich F., Heimberg R., & Gross J. J. (2016). Group CBT versus MBSR for social anxiety disorder: A randomized controlled trial. Journal of Consulting and Clinical Psychology, 84, 427–437. 10.1037/ccp0000092 26950097 PMC4837056

[c28] Goldin P., Ziv M., Jazaieri H., & Gross J. J. (2012). Randomized controlled trial of mindfulness-based stress reduction versus aerobic exercise: Effects on the self-referential brain network in social anxiety disorder. Frontiers in Human Neuroscience, 6, 295 10.3389/fnhum.2012.00295 23133411 PMC3488800

[c29] Gross C. R., Kreitzer M. J., Reilly-Spong M., Wall M., Winbush N. Y., Patterson R., . . . Cramer-Bornemann M. (2011). Mindfulness-based stress reduction versus pharmacotherapy for chronic primary insomnia: A randomized controlled clinical trial. Explore, 7, 76–87. 10.1016/j.explore.2010.12.003 21397868 PMC3077056

[c30] Gross C. R., Kreitzer M. J., Thomas W., Reilly-Spong M., Cramer-Bornemann M., Nyman J. A., . . . Ibrahim H. N. (2010). Mindfulness-based stress reduction for solid organ transplant recipients: A randomized controlled trial. Alternative Therapies in Health and Medicine, 16, 30–38. PMC3076132 20882729

[c84] Gross J. J., & John O. P. (2003). Individual differences in two emotion regulation processes: Implications for affect, relationships, and wellbeing. Journal of Personality and Social Psychology, 85, 348–362. 12916575 10.1037/0022-3514.85.2.348

[c31] Gu J., Strauss C., Bond R., & Cavanagh K. (2015). How do mindfulness-based cognitive therapy and mindfulness-based stress reduction improve mental health and wellbeing? A systematic review and meta-analysis of mediation studies. Clinical Psychology Review, 37, 1–12. 10.1016/j.cpr.2015.01.006 25689576

[c82] Gu J., Strauss C., Crane C., Barnhofer T., Karl A., Cavanagh K., & Kuyken W. (2016). Examining the factor structure of the 39-item and 15-item versions of the Five Facet Mindfulness Questionnaire before and after mindfulness-based cognitive therapy for people with recurrent depression. Psychological Assessment, 28, 791–802. 27078186 10.1037/pas0000263 PMC4928699

[c32] Hayes A. M., & Feldman G. (2004). Clarifying the construct of mindfulness in the context of emotion regulation and the process of change in therapy. Clinical Psychology: Science and Practice, 11, 255–262. 10.1093/clipsy.bph080

[c33] Higgins J. P., Altman D. G., Gøtzsche P. C., Jüni P., Moher D., Oxman A. D., . . . the Cochrane Bias Methods Group, & the Cochrane Statistical Methods Group (2011). The Cochrane Collaboration’s tool for assessing risk of bias in randomised trials. British Medical Journal, 343, d5928 10.1136/bmj.d5928 22008217 PMC3196245

[c34] Higgins J. P., & Green S. (2011). Cochrane handbook for systematic reviews of interventions (Version 5.1.0). Oxford, United Kingdom: The Cochrane Collaboration.

[c35] Higgins J. P., & Thompson S. G. (2002). Quantifying heterogeneity in a meta-analysis. Statistics in Medicine, 21, 1539–1558. 10.1002/sim.1186 12111919

[c36] Hoge E. A., Bui E., Goetter E., Robinaugh D. J., Ojserkis R. A., Fresco D. M., & Simon N. M. (2015). Change in decentering mediates improvement in anxiety in mindfulness-based stress reduction for generalized anxiety disorder. Cognitive Therapy and Research, 39, 228–235. 10.1007/s10608-014-9646-4 28316355 PMC5354303

[c37] Hou J., Wong S. Y., Lo H. H., Mak W. W., & Ma H. S. (2014). Validation of a Chinese version of the Five Facet Mindfulness Questionnaire in Hong Kong and development of a short form. Assessment, 21, 363–371. 10.1177/1073191113485121 23596271

[c39] Huijbers M. J., Spinhoven P., Spijker J., Ruhé H. G., van Schaik D. J., van Oppen P., . . . Speckens A. E. (2015). Adding mindfulness-based cognitive therapy to maintenance antidepressant medication for prevention of relapse/recurrence in major depressive disorder: Randomised controlled trial. Journal of Affective Disorders, 187, 54–61. 10.1016/j.jad.2015.08.023 26318271

[c41] James K., & Rimes K. A. (2018). Mindfulness-based cognitive therapy versus pure cognitive behavioural self-help for perfectionism: A pilot randomised study. Mindfulness, 9, 801–814. 29875882 10.1007/s12671-017-0817-8 PMC5968046

[c42] Jedel S., Merriman P., Hoffman A., Swanson B., Fogg L., & Keshavarzian A. (2013). Relationship of mindfulness, quality of life, and psychiatric symptoms among patients with ulcerative colitis. Mindfulness, 4, 296–300. 10.1007/s12671-012-0128-z

[c43] Jensen C. G., Krogh S. C., Westphael G., & Hjordt L. V. (2019). Mindfulness is positively related to socioeconomic job status and income and independently predicts mental distress in a long-term perspective: Danish validation studies of the Five-Factor Mindfulness Questionnaire. Psychological Assessment, 31, e1–e20. 30520656 10.1037/pas0000667

[c44] Jensen C. G., Niclasen J., Vangkilde S. A., Petersen A., & Hasselbalch S. G. (2016). General inattentiveness is a long-term reliable trait independently predictive of psychological health: Danish validation studies of the Mindful Attention Awareness Scale. Psychological Assessment, 28, e70–e87. 10.1037/pas0000196 26751089

[c45] Jensen C. G., Vangkilde S., Frokjaer V., & Hasselbalch S. G. (2012). Mindfulness training affects attention—or is it attentional effort? Journal of Experimental Psychology: General, 141, 106–123. 10.1037/a0024931 21910559

[c46] Johns S. A., Von Ah D., Brown L. F., Beck-Coon K., Talib T. L., Alyea J. M., . . . Giesler R. B. (2016). Randomized controlled pilot trial of mindfulness-based stress reduction for breast and colorectal cancer survivors: Effects on cancer-related cognitive impairment. Journal of Cancer Survivorship, 10, 437–448. 10.1007/s11764-015-0494-3 26586494 PMC4864185

[c47] Kabat-Zinn J. (1982). An outpatient program in behavioral medicine for chronic pain patients based on the practice of mindfulness meditation: Theoretical considerations and preliminary results. General Hospital Psychiatry, 4, 33–47. 10.1016/0163-8343(82)90026-3 7042457

[c86] Kabat-Zinn J. (1994). Wherever you go, there you are: Mindfulness meditation in everyday life. New York, NY: Hyperion.

[c87] Kabat-Zinn J. (2003). Mindfulness-based interventions in context: Past, present and future. Clinical Psychology: Science and Practice, 10, 144–156.

[c48] Khoury B., Lecomte T., Fortin G., Masse M., Therien P., Bouchard V., . . . Hofmann S. G. (2013). Mindfulness-based therapy: A comprehensive meta-analysis. Clinical Psychology Review, 33, 763–771. 10.1016/j.cpr.2013.05.005 23796855

[c49] Kuyken W., Byford S., Taylor R. S., Watkins E., Holden E., White K., . . . Teasdale J. D. (2008). Mindfulness-based cognitive therapy to prevent relapse in recurrent depression. Journal of Consulting and Clinical Psychology, 76, 966–978. 10.1037/a0013786 19045965

[c50] Kuyken W., Hayes R., Barrett B., Byng R., Dalgleish T., Kessler D., . . . Byford S. (2015). Effectiveness and cost-effectiveness of mindfulness-based cognitive therapy compared with maintenance antidepressant treatment in the prevention of depressive relapse or recurrence (PREVENT): A randomised controlled trial. The Lancet, 386, 63–73. 10.1016/S0140-6736(14)62222-4 25907157

[c51] Lau M. A., Bishop S. R., Segal Z. V., Buis T., Anderson N. D., Carlson L., . . . Devins G. (2006). The Toronto Mindfulness Scale: Development and validation. Journal of Clinical Psychology, 62, 1445–1467. 10.1002/jclp.20326 17019673

[c52] Linehan M. M. (1993). Cognitive-behavioral treatment of borderline personality disorder. New York, NY: Guilford Press.

[c88] Linehan M. M. (2015). DBT skills training manual (2nd ed.). New York, NY: Guilford.

[c53] Lipsey M. W. (2003). Those confounded moderators in meta-analysis: Good, bad, and ugly. The Annals of the American Academy of Political and Social Science, 587, 69–81. 10.1177/0002716202250791

[c54] MacCoon D. G., Imel Z. E., Rosenkranz M. A., Sheftel J. G., Weng H. Y., Sullivan J. C., . . . Lutz A. (2012). The validation of an active control intervention for Mindfulness Based Stress Reduction (MBSR). Behaviour Research and Therapy, 50, 3–12. 10.1016/j.brat.2011.10.011 22137364 PMC3257026

[c55] Mallya S., & Fiocco A. (2016). Effects of mindfulness training on cognition and well-being in healthy older adults. Mindfulness, 7, 453–465. 10.1007/s12671-015-0468-6

[c56] Manicavasagar V., Perich T., & Parker G. (2012). Cognitive predictors of change in cognitive behaviour therapy and mindfulness-based cognitive therapy for depression. Behavioural and Cognitive Psychotherapy, 40, 227–232. 10.1017/S1352465811000634 22017810

[c90] Marlatt G. A., & Kristeller J. L. (1999). Mindfulness and meditation In Miller W. R. (Ed.), Integrating spirituality into treatment (pp. 67–84). Washington, DC: American Psychological Association.

[c57] McKenna L., Marks E. M., Hallsworth C. A., & Schaette R. (2017). Mindfulness-based cognitive therapy as a treatment for chronic tinnitus: A randomized controlled trial. Psychotherapy and Psychosomatics, 86, 351–361. 10.1159/000478267 29131084

[c91] Moher D., Liberati A., Tetzlaff J., & Altman D. G. (2009). Preferred reporting items for systematic reviews and meta-analyses: the PRISMA statement. British Medical Journal, 339, b2535. 19622551 10.1136/bmj.b2535 PMC2714657

[c58] Morone N. E., Greco C. M., Moore C. G., Rollman B. L., Lane B., Morrow L. A., . . . Weiner D. K. (2016). A mind-body program for older adults with chronic low back pain: A randomized clinical trial. Journal of the American Medical Association Internal Medicine, 176, 329–337. 10.1001/jamainternmed.2015.8033 26903081 PMC6361386

[c85] Morrison A. S., Potter C. M., Carper M. M., Kinner D. G., Jensen D., Bruce L., . . . Heimberg R. G. (2015). The cognitive distortions questionnaire (CD-Quest): Psychometric properties and exploratory factor analysis. International Journal of Cognitive Therapy, 8, 287–305.

[c59] Park T., Reilly-Spong M., & Gross C. R. (2013). Mindfulness: A systematic review of instruments to measure an emergent patient-reported outcome (PRO). Quality of Life Research: An International Journal of Quality of Life Aspects of Treatment, Care & Rehabilitation, 22, 2639–2659. 10.1007/s11136-013-0395-8 PMC3745812 23539467

[c60] Pbert L., Madison J. M., Druker S., Olendzki N., Magner R., Reed G., . . . Carmody J. (2012). Effect of mindfulness training on asthma quality of life and lung function: A randomised controlled trial. Thorax, 67, 769–776. 10.1136/thoraxjnl-2011-200253 22544892 PMC4181405

[c61] Polusny M. A., Erbes C. R., Thuras P., Moran A., Lamberty G. J., Collins R. C., . . . Lim K. O. (2015). Mindfulness-based stress reduction for posttraumatic stress disorder among veterans: A randomized clinical trial. Journal of the American Medical Association, 314, 456–465. 10.1001/jama.2015.8361 26241597

[c62] Quaglia J. T., Braun S. E., Freeman S. P., McDaniel M. A., & Brown K. W. (2016). Meta-analytic evidence for effects of mindfulness training on dimensions of self-reported dispositional mindfulness. Psychological Assessment, 28, 803–818. 10.1037/pas0000268 27078183

[c63] Raja-Khan N., Agito K., Shah J., Stetter C. M., Gustafson T. S., Socolow H., . . . Legro R. S. (2017). Mindfulness-based stress reduction in women with overweight or obesity: A randomized clinical trial. Obesity, 25, 1349–1359. 10.1002/oby.21910 28686006 PMC5529243

[c64] Rosenberg M. S. (2005). The file-drawer problem revisited: A general weighted method for calculating fail-safe numbers in meta-analysis. Evolution: International Journal of Organic Evolution, 59, 464–468. 10.1111/j.0014-3820.2005.tb01004.x 15807430

[c65] Rosenthal R. (1979). The file drawer problem and tolerance for null results. Psychological Bulletin, 86, 638–641. 10.1037/0033-2909.86.3.638

[c66] Schmidt S., Grossman P., Schwarzer B., Jena S., Naumann J., & Walach H. (2011). Treating fibromyalgia with mindfulness-based stress reduction: Results from a 3-armed randomized controlled trial. Pain, 152, 361–369. 10.1016/j.pain.2010.10.043 21146930

[c67] Segal Z. V., Bieling P., Young T., MacQueen G., Cooke R., Martin L., . . . Levitan R. D. (2010). Antidepressant monotherapy vs sequential pharmacotherapy and mindfulness-based cognitive therapy, or placebo, for relapse prophylaxis in recurrent depression. Archives of General Psychiatry, 67, 1256–1264. 10.1001/archgenpsychiatry.2010.168 21135325 PMC3311113

[c68] Segal Z. V., Williams J. M. G., & Teasdale J. D. (2013). Mindfulness-based cognitive therapy for depression (2nd ed.). New York, NY: Guilford Press.

[c89] Simshauser K., Luking M., Kaube H., Schultz C., & Schmidt S. (in press). Is mindfulness-based stress reduction (MBSR) a promising and feasible intervention for patients suffering from migraine? A randomized controlled pilot trial. Complementary Medicine Research. 10.1159/000501425 31390617

[c69] Shallcross A. J., Gross J. J., Visvanathan P. D., Kumar N., Palfrey A., Ford B. Q., . . . Mauss I. B. (2015). Relapse prevention in major depressive disorder: Mindfulness-based cognitive therapy versus an active control condition. Journal of Consulting and Clinical Psychology, 83, 964–975. 10.1037/ccp0000050 26371618 PMC4571290

[c71] Trousselard M., Steiler D., Raphel C., Cian C., Duymedjian R., Claverie D., & Canini F. (2010). Validation of a French version of the Freiburg Mindfulness Inventory - short version: Relationships between mindfulness and stress in an adult population. BioPsychoSocial Medicine, 4, 8 10.1186/1751-0759-4-8 20704696 PMC2927476

[c72] Vidrine J. I., Spears C. A., Heppner W. L., Reitzel L. R., Marcus M. T., Cinciripini P. M., . . . Wetter D. W. (2016). Efficacy of mindfulness-based addiction treatment (MBAT) for smoking cessation and lapse recovery: A randomized clinical trial. Journal of Consulting and Clinical Psychology, 84, 824–838. 10.1037/ccp0000117 27213492 PMC5061584

[c73] Visted E., Vollestad J., Nielsen M., & Nielsen G. (2015). The impact of group-based mindfulness training on self-reported mindfulness: A systematic review and meta-analysis. Mindfulness, 6, 501–522. 10.1007/s12671-014-0283-5

[c74] Wetherell J. L., Hershey T., Hickman S., Tate S. R., Dixon D., Bower E. S., & Lenze E. J. (2017). Mindfulness-based stress reduction for older adults with stress disorders and neurocognitive difficulties: A randomized controlled trial. The Journal of Clinical Psychiatry, 78, e734–e743. 10.4088/JCP.16m10947 28686822

[c75] Williams J. M. G., Crane C., Barnhofer T., Brennan K., Duggan D. S., Fennell M. J., . . . Russell I. T. (2014). Mindfulness-based cognitive therapy for preventing relapse in recurrent depression: A randomized dismantling trial. Journal of Consulting and Clinical Psychology, 82, 275–286. 10.1037/a0035036 24294837 PMC3964149

[c76] Williams M. J., Dalgleish T., Karl A., & Kuyken W. (2014). Examining the factor structures of the Five Facet Mindfulness Questionnaire and the Self-Compassion Scale. Psychological Assessment, 26, 407–418. 10.1037/a0035566 24490681

[c77] Witkiewitz K., Warner K., Sully B., Barricks A., Stauffer C., Thompson B. L., & Luoma J. B. (2014). Randomized trial comparing mindfulness-based relapse prevention with relapse prevention for women offenders at a residential addiction treatment center. Substance Use & Misuse, 49, 536–546. 10.3109/10826084.2013.856922 24611849

[c78] Wong S. Y., Yip B. H., Mak W. W., Mercer S., Cheung E. Y., Ling C. Y., . . . Ma H. S. (2016). Mindfulness-based cognitive therapy v. group psychoeducation for people with generalised anxiety disorder: Randomised controlled trial. The British Journal of Psychiatry, 209, 68–75. 10.1192/bjp.bp.115.166124 26846612

