# Correction to Baer et al. (2019)

**DOI:** 10.1037/pas0000777

**Published:** 2019-10

The article “Differential Sensitivity of Mindfulness Questionnaires to Change With Treatment: A Systematic Review and Meta-Analysis,” by Ruth Baer, Jenny Gu, Kate Cavanagh, and Clara Strauss (*Psychological Assessment*, 2019, Vol. 31, No. 10, pp. 1247–1263, http://dx.doi.org/10.1037/pas0000744), should have been published under the terms of the Creative Commons Attribution License (CC BY 3.0). Therefore, the article was amended to list the authors as copyright holders, and information about the terms of the CC BY 3.0 was added to the author note. In addition, the article is now open access. All versions of this article have been corrected.

