# Supplementary material for: Differential Sensitivity of Mindfulness Questionnaires to Change With Treatment: A Systematic Review and Meta-Analysis
Source: Psychol Assess. 2019 Aug 1;31(10):1247–63. doi: 10.1037/pas0000744 (PMC6793937; doi:10.1037/pas0000744)
Supplement: Supplementary file 1 [file PAS-2018-2228_Suppl.docx]

**Supplementary Materials** for:

Discriminant Validity of Mindfulness Questionnaires: A Meta-Analytic Review of Differential Sensitivity to Change with Intervention


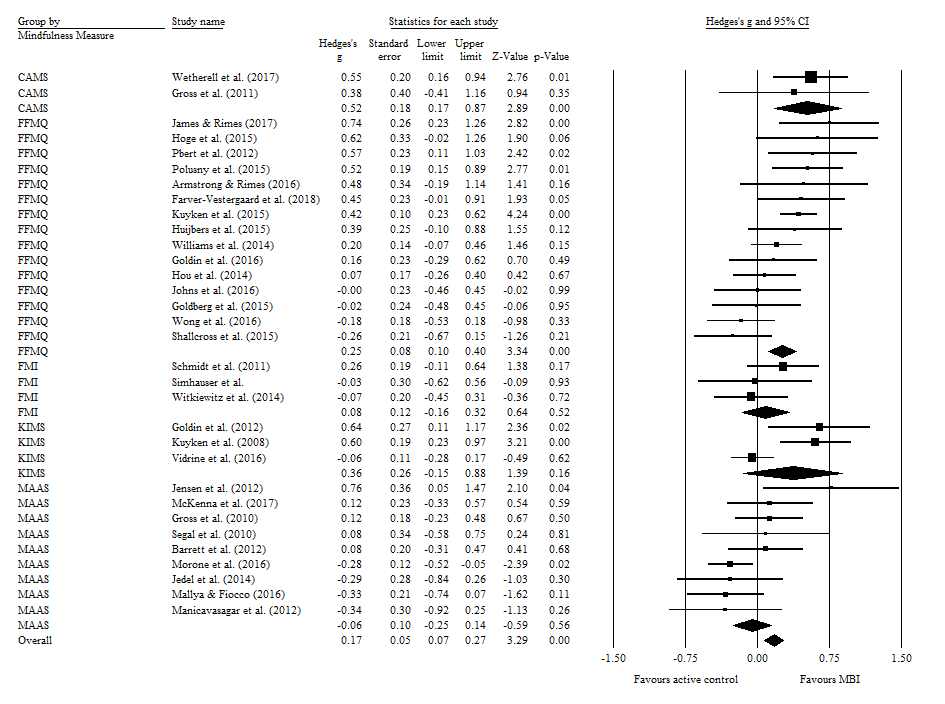


*Figure 1.* Forest plot of the effect of MBIs compared to active control conditions on pre-post change in total mindfulness as a function of mindfulness measure (FFMQ, MAAS, FMI, KIMS, CAMS-R).

*
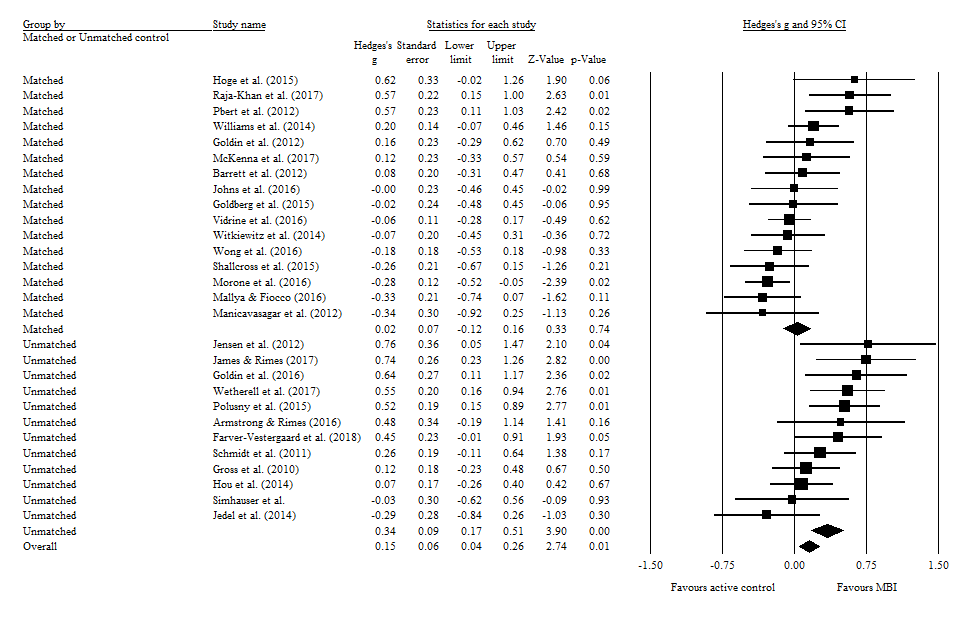
*

*Figure 2.* Forest plot of the effect of MBIs compared to active control conditions on pre-post change in total mindfulness as a function of MBI and control condition matching for session time and structure.

*
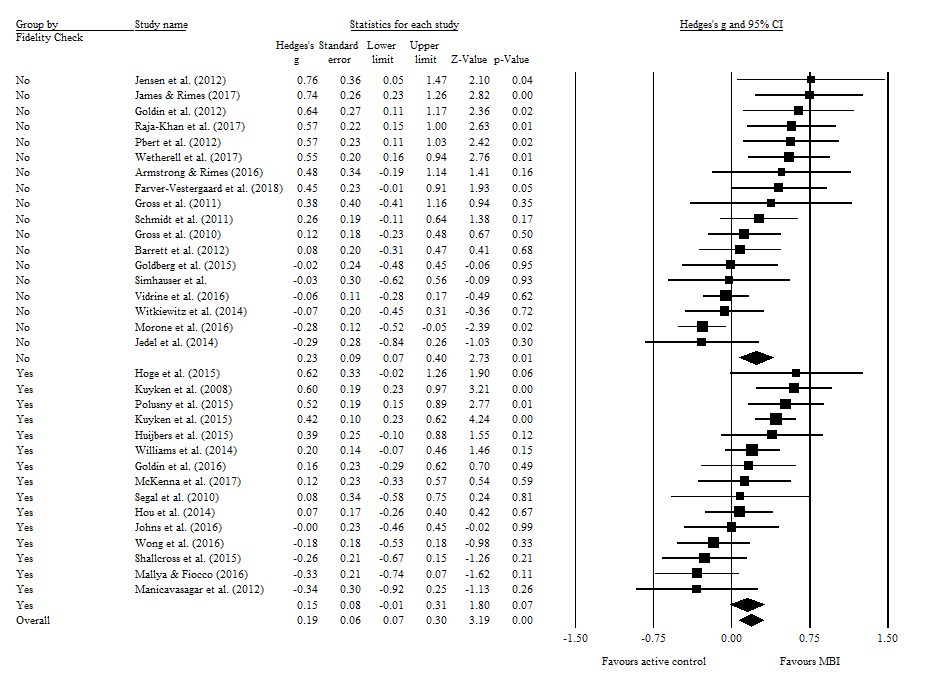
*

*Figure 3.* Forest plot of the effect of MBIs compared to active control conditions on pre-post change in total mindfulness as a function of fidelity to the MBI.

*
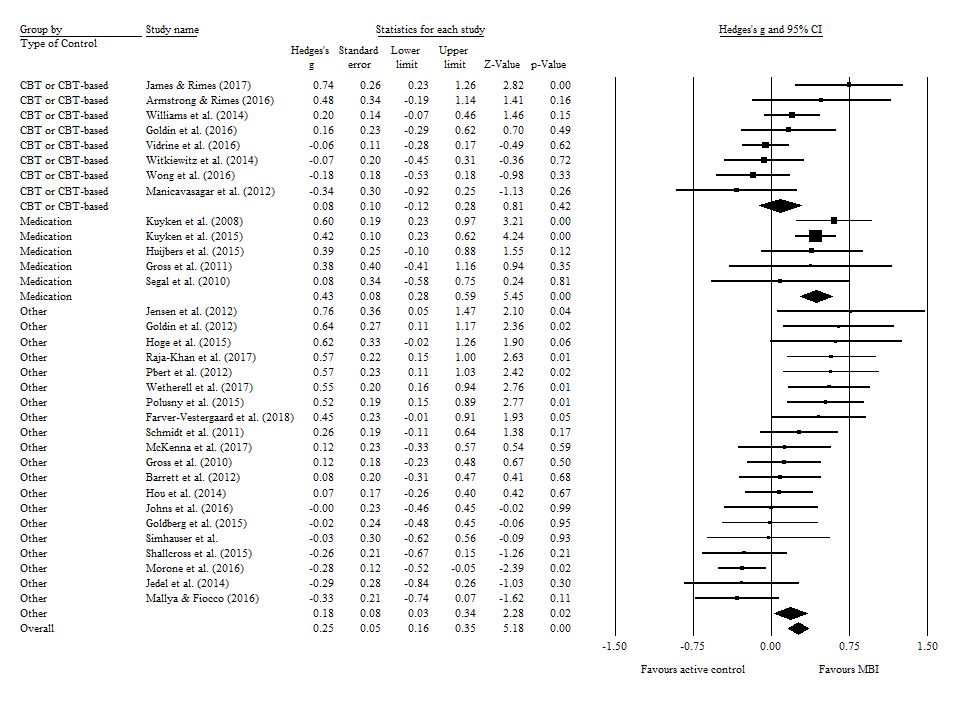
*

*Figure 4.* Forest plot of the effect of MBIs compared to active control conditions on pre-post change in total mindfulness as a function of type of control condition (CBT/CBT-based, medication, other).

*
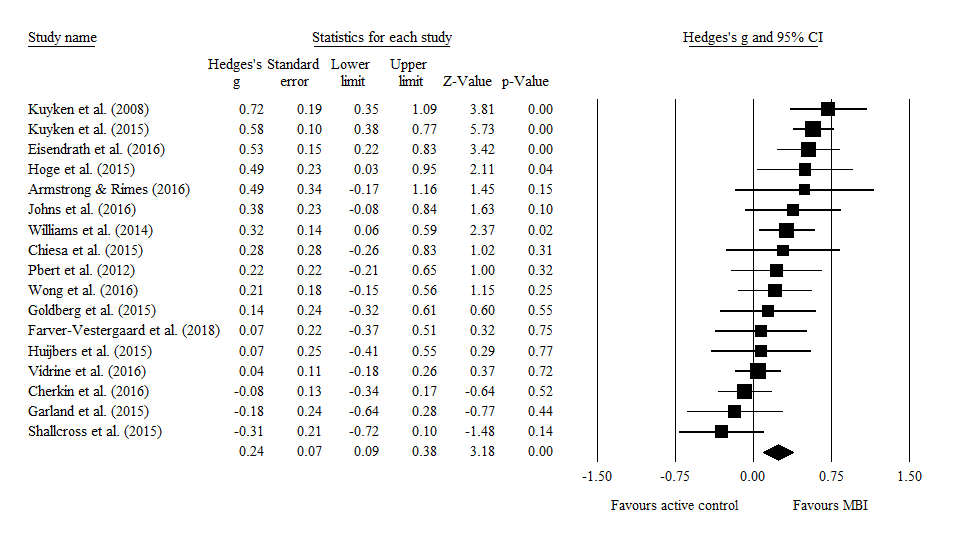
*

*Figure 5.* Forest plot of the effect of MBIs compared to active control conditions on pre-post change in FFMQ/KIMS observing.

*
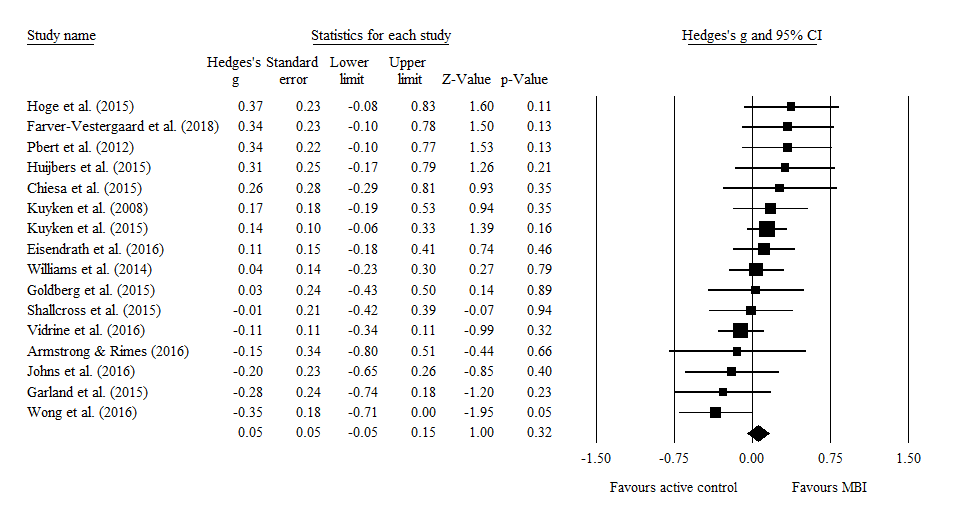
*

*Figure 6.* Forest plot of the effect of MBIs compared to active control conditions on pre-post change in FFMQ/KIMS describing.

*
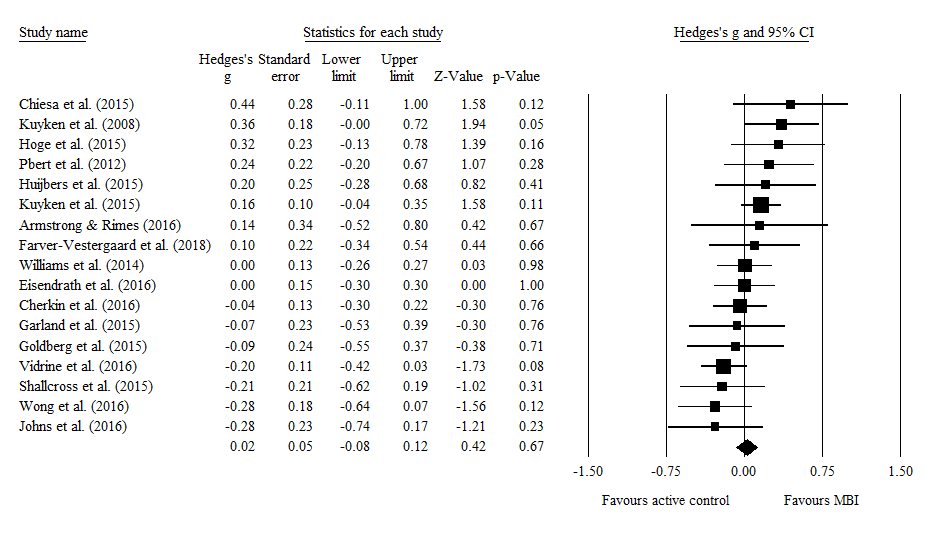
*

*Figure 7*. Forest plot of the effect of MBIs compared to active control conditions on pre-post change in FFMQ/KIMS acting with awareness.

*
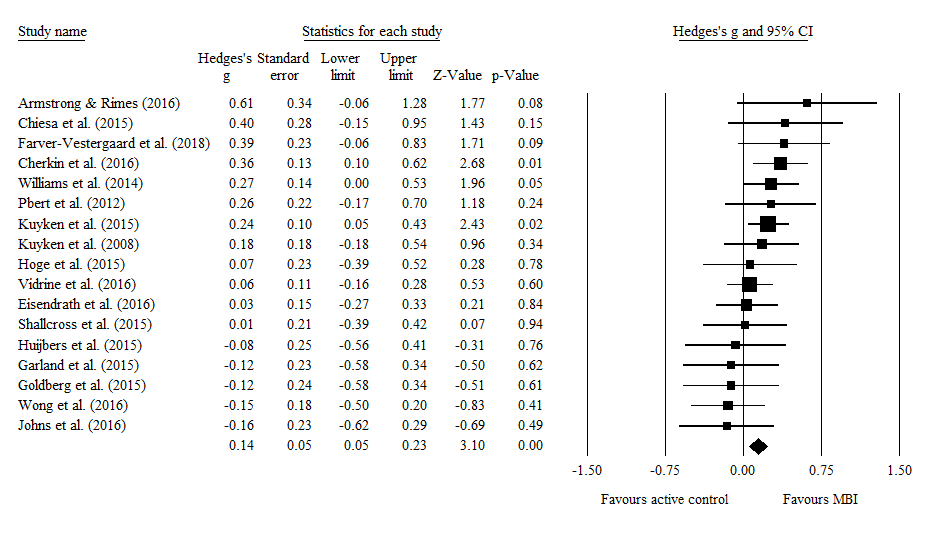
*

*Figure 8.* Forest plot of the effect of MBIs compared to active control conditions on pre-post change in FFMQ/KIMS non-judging.

*
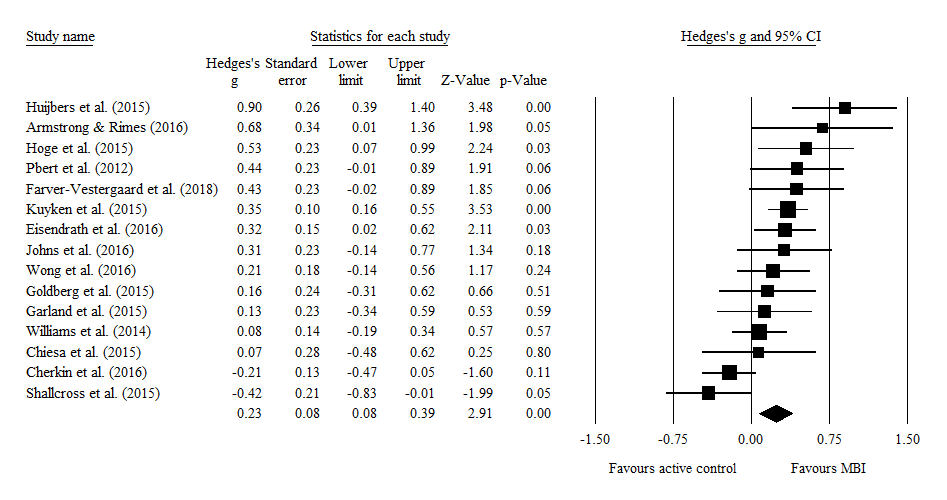
*

*Figure 9.* Forest plot of the effect of MBIs compared to active control conditions on pre-post change in FFMQ non-reactivity.


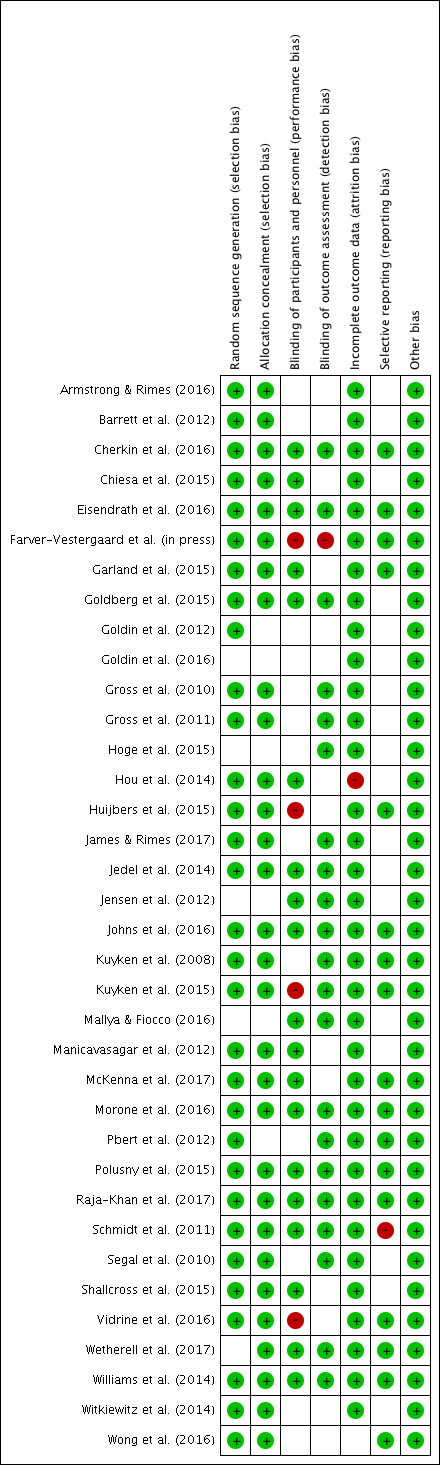


*Figure 10.* Risk of bias for each criterion for each study. Green circles indicate low risk of bias, white spaces indicate unclear risk of bias, and red circles indicate high risk of bias.

*
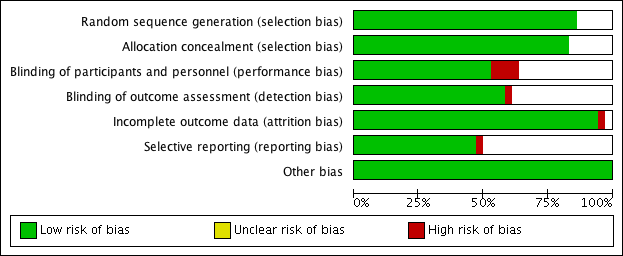
 Figure 11.* Percentages of studies with low, unclear, and high risk of bias for each criterion. Green indicates low risk of bias, white indicates unclear risk of bias, and red indicates high risk of bias.

Table 1.

Quality scores for included published studies based on risk of bias (*N* = 36).

| Study | Random sequence generation | Allocation concealment | Blinding of participants and personnel | Blinding of outcome assessment | Completeness of outcome data | Selective outcome reporting | Other bias | Total quality score based on risk of bias (/7) |
| --- | --- | --- | --- | --- | --- | --- | --- | --- |
| Armstrong & Rimes (2016) | 1 | 1 | 0 (unclear risk) | 0 (unclear risk) | 1 | 0 (unclear risk) | 1 | 4 |
| Barrett et al. (2012) | 1 | 1 | 0 (unclear risk) | 0 (unclear risk) | 1 | 0 (unclear risk) | 1 | 4 |
| Cherkin et al. (2016) | 1 | 1 | 1 | 1 | 1 | 1 | 1 | 7 |
| Chiesa et al. (2015) | 1 | 1 | 1 | 0 (unclear risk) | 1 | 0 (unclear risk) | 1 | 5 |
| Eisendrath et al. (2016) | 1 | 1 | 1 | 1 | 1 | 1 | 1 | 7 |
| Farver-Vestergaard et al. (2018) | 1 | 1 | 0 | 0 | 1 | 1 | 1 | 5 |
| Garland et al. (2015) | 1 | 1 | 1 | 0 (unclear risk) | 1 | 1 | 1 | 6 |
| Goldberg et al. (2015) | 1 | 1 | 1 | 1 | 1 | 0 (unclear risk) | 1 | 6 |
| Goldin et al. (2012) | 1 | 0 (unclear risk) | 0 (unclear risk) | 0 (unclear risk) | 1 | 0 (unclear risk) | 1 | 3 |
| Goldin et al. (2016) | 0 (unclear risk) | 0 (unclear risk) | 0 (unclear risk) | 0 (unclear risk) | 1 | 0 (unclear risk) | 1 | 2 |
| Gross et al. (2010) | 1 | 1 | 0 (unclear risk) | 1 | 1 | 0 (unclear risk) | 1 | 5 |
| Gross et al. (2011) | 1 | 1 | 0 (unclear risk) | 1 | 1 | 0 (unclear risk) | 1 | 5 |
| Hoge et al. (2015) | 0 (unclear risk) | 0 (unclear risk) | 0 (unclear risk) | 1 | 1 | 0 (unclear risk) | 1 | 3 |
| Hou et al. (2014) | 1 | 1 | 1 | 0 (unclear risk) | 0 | 0 (unclear risk) | 1 | 4 |
| Huijbers et al. (2015) | 1 | 1 | 0 | 0 (unclear risk) | 1 | 1 | 1 | 5 |
| James & Rimes (2017) | 1 | 1 | 0 (unclear risk) | 1 | 1 | 0 (unclear risk) | 1 | 5 |
| Jedel et al. (2014) | 1 | 1 | 1 | 1 | 1 | 0 (unclear risk) | 1 | 6 |
| Jensen et al. (2012) | 0 (unclear risk) | 0 (unclear risk) | 1 | 1 | 1 | 0 (unclear risk) | 1 | 4 |
| Johns et al. (2016) | 1 | 1 | 1 | 1 | 1 | 1 | 1 | 7 |
| Kuyken et al. (2008) | 1 | 1 | 0 (unclear risk) | 1 | 1 | 1 | 1 | 6 |
| Kuyken et al. (2015) | 1 | 1 | 0 | 1 | 1 | 1 | 1 | 6 |
| Mallya & Fiocco (2016) | 0 (unclear risk) | 0 (unclear risk) | 1 | 1 | 1 | 0 (unclear risk) | 1 | 4 |
| Manicavasagar et al. (2012) | 1 | 1 | 1 | 0 (unclear risk) | 1 | 0 (unclear risk) | 1 | 5 |
| McKenna et al. (2017) | 1 | 1 | 1 | 0 (unclear risk) | 1 | 1 | 1 | 6 |
| Morone et al. (2016) | 1 | 1 | 1 | 1 | 1 | 1 | 1 | 7 |
| Pbert et al. (2012) | 1 | 0 (unclear risk) | 0 (unclear risk) | 1 | 1 | 1 | 1 | 5 |
| Polusny et al. (2015) | 1 | 1 | 1 | 1 | 1 | 1 | 1 | 7 |
| Raja-Khan et al. (2017) | 1 | 1 | 1 | 1 | 1 | 1 | 1 | 7 |
| Schmidt et al. (2011) | 1 | 1 | 1 | 1 | 1 | 0 | 1 | 6 |
| Segal et al. (2010) | 1 | 1 | 0 (unclear risk) | 1 | 1 | 0 (unclear risk) | 1 | 5 |
| Shallcross et al. (2015) | 1 | 1 | 1 | 0 (unclear risk) | 1 | 0 (unclear risk) | 1 | 5 |
| Vidrine et al. (2016) | 1 | 1 | 0 | 0 (unclear risk) | 1 | 1 | 1 | 5 |
| Wetherell et al. (2017) | 0 (unclear risk) | 1 | 1 | 1 | 1 | 1 | 1 | 6 |
| Williams et al. (2014) | 1 | 1 | 1 | 1 | 1 | 1 | 1 | 7 |
| Witkiewitz et al. (2014) | 1 | 1 | 0 (unclear risk) | 0 (unclear risk) | 1 | 0 (unclear risk) | 1 | 4 |
| Wong et al. (2016) | 1 | 1 | 0 (unclear risk) | 0 (unclear risk) | 0 (unclear risk) | 1 | 1 | 4 |
